# Synthesis and Evaluation of C15 Triene Urushiol Derivatives as Potential Anticancer Agents and HDAC2 Inhibitor

**DOI:** 10.3390/molecules23051074

**Published:** 2018-05-03

**Authors:** Zhiwen Qi, Chengzhang Wang, Jianxin Jiang

**Affiliations:** 1 Institute of Chemical Industry of Forest Products, CAF, Nanjing 210042, China; angelkissgod@163.com; 2 Key and Open Laboratory on Forest Chemical Engineering, SFA, Nanjing 210042, China; 3 College of Material Science and Technology, Beijing Forestry University, Beijing 100083, China; jiangjx@bjfu.edu.cn

**Keywords:** urushiol, derivatives, antitumor, HDAC2, molecular docking

## Abstract

A series of C15 triene urushiol derivatives were synthesized and evaluated for their anti-HepG2 aggregation in vitro. The results indicated that all compounds had an effective anti-HepG2 vitality. Compound **1** was a potent inhibitor of HepG2 with IC_50_ of 7.886 μM and 150 μM against LO2. Moreover, compound **1** increased the apoptosis of HepG2. Compound **1**’s thiol sulfur formed hydrogen bonding interactions with Gly154 and Tyr308, respectively, and made it bound more closely to HDAC2. In addition, it also formed hydrophobic interactions with the residues His33, Pro106, Val107, Gly154, Phe155, and His183, and was provided with a strong van der Waals force by the hydrophobic action.

## 1. Introduction

As a natural alkyl phenol, urushiol is similar to SAHA which is efficient for tumor HDACs, but has mutagenic, carcinogenic and genotoxic potential [1]. Urushiol is able to modulate the activity of SIRT inhibitors [2] and prevent lung cancer cell-A549 proliferation [3]. It has a good antibacterial effect on *Helicobacter pylori* [4,5,6], and stimulates the activity of certain cells of mice, reducing the risk of fatty liver in mice and changing microbial morphology [5,7].

Our research has indicated that the urushiol derivatives have excellent binding force to HDAC2 [8,9], which are a group of zinc metalloenzymes, regulating chromatin remodeling and gene transcription by catalyzing the removal of an acetyl group moiety from the ε-amino groups of lysine residues on the amino terminal tails of the core histones [10]. HDACs play a pivotal role in the regulation of gene expression, cell growth, and proliferation [11]. Overexpression of HDACs has been linked to the development of cancers in humans [12]. This evidence suggests that urushiol has potential antitumor effect.

Modification is a usual method to improve product qualities. For example, common catechols are mainly modified by methylation (sulfonation) [13], halogenation [14,15], Pechmann reaction [16,17,18], and boric esterification [19] which is moderately stable in air [20] due to its high affinity for diols [21], and is frequently used for recognizing carbohydrate [22,23,24]. Besides, some highly fluorinated catechol compounds have excellent cytotoxic activity against prostate cancer cells, and decrease their viability [25], giving rise to a gene delivery ability [26]. They also have excellent organic electronics property [27], and are used as probes [28].

Hence, based on clinical molecule simulation principle [8], we tried to lower the urushiol toxicity and enhance its efficacy. In this study, we chemically modified the C15 triene urushiol to reduce its toxicity by applying the strategies mentioned above. The best bioactive compound to inhibit HepG2 was evaluated by FCM-flow cytometry, Western blot and molecule docking analysis.

## 2. Results

### 2.1. Chemistry

**Scheme 1:**
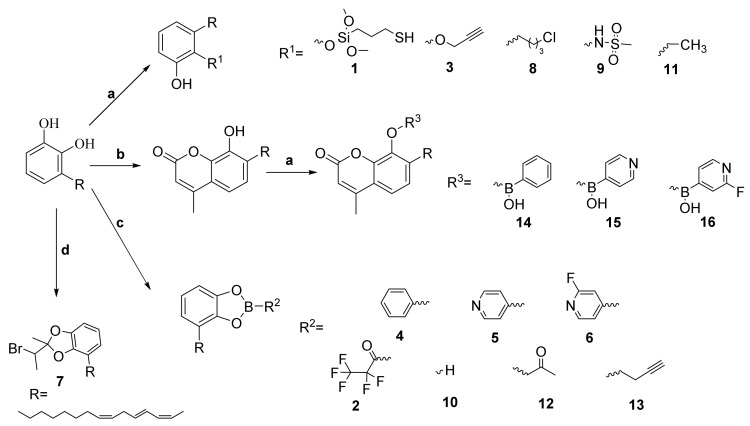
Synthetic routes for C15 triene urushiol derivatives via phenol hydroxide protection. Reagents and conditions: (**a**) K_2_CO_3_, acetone, reflux, 24 h; (**b**) *p*-Toluene, ethyl acetoacetate, r.t., 8 h; and (**c**) DCM/EA, 60 °C, 2 h, in a pressure tube.

C15 triene urushiol was extracted and isolated from the lacquer (*Toxicodendron vernicifluum*), and purified by silica gel column chromatography. According to the general pathway outlined in Scheme 1, target compounds **1**–**16** mentioned in this research were synthesized in a one-step reaction. Since the ionization constant of 2-position phenolic hydroxyl is ten times higher than that of 1-position of benzene ring [29], the halogenation reaction of phenol hydroxyl group at 2-position is more likely to take place under the condition of alkalinity such as K_2_CO_3_, and produce compounds **1**, **3**, **8**, **9**, and **11**. The phenolic hydroxyl groups of urushiol Pechmann derivative are easy to react with similar reaction to form compounds **14**, **15** and **16**. In addition, the boric acid esterification reaction between the two phenolic hydroxyl groups of urushiol is easy to occur, forming compounds **2**, **4**, **5**, **6**, **10**, **12** and **13**. The detailed synthetic processes are described in the Materials and Methods Section. Compounds **1**–**16** were purified by silica gel column chromatography and their chemical structures were characterized by using ^1^H NMR, ^13^C NMR, and ESI-MS.

### 2.2. Anti-Tumor Activity

**Figure 1:**
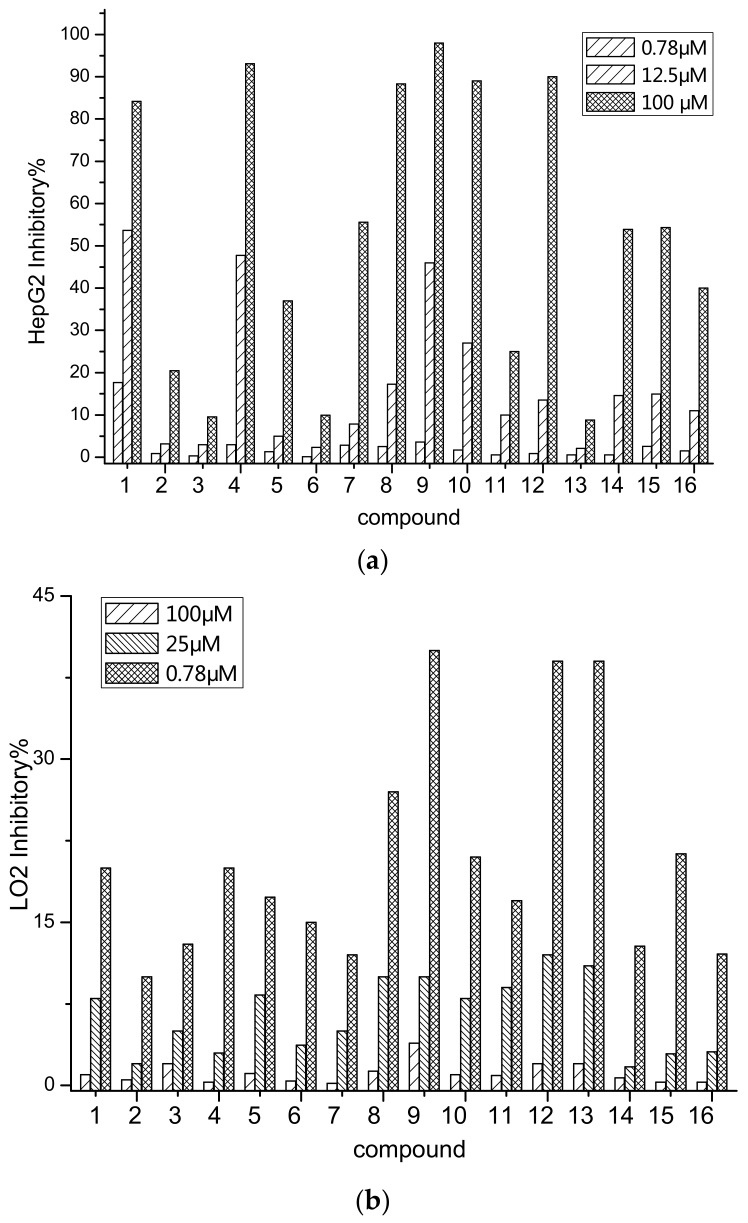
Compounds **1**–**16** inhibitory on HepG2 and LO2 in vitro: (**a**) the inhibitory rate on HepG2; and (**b**) the inhibitory rate on LO2. Statistical treatment: SPSS 17.0 Statistical software for data processing and statistical scoring Analysis. The experimental data are expressed as mean ± standard deviation (mean ± SD). Normality and variance homogeneity test are carried out to satisfy the normal distribution and variance. Univariate ANOVA was used to compare the homogeneity. *p* < 0.05. There was significant difference between groups.

As shown in Figure 1 and Table 1, compounds **1**–**16** were evaluated for their inhibitory effects on the proliferation of HepG2 and LO_2_ cells by MTT assay. The structure–activity relationship of the above-mentioned urushiol derivatives showed that, with the thiol-containing long-chain alkane, phenylboronic acid and amino sulfoxide, urushiol as well as urushiol Pechmann derivative played an effective role in attenuating and increasing the efficiency. It provided the experimental basis for the subsequent synthesis of attenuating and improving urushiol derivatives.

We found that the inhibitory activity of compounds **1**, **4** and **9** on HepG2 cells proliferation was strong. The IC_50_ values of all compounds were obtained in gradient experiment. The inhibitory activity of compound **1** on HepG2 cells was 7.886 μM.

Compounds **1**, **4**, and **9**, respectively, introduced a thiol group on the 2-position phenolic hydroxyl group, a benzene borate bond, and an amino sulfoxide, which increased their anti-HepG2 activity to 7–15 μM and reduced the sensitization of urushiol. Introducing a long-chain alkane chloride into the phenolic hydroxyl group of 2-position urushiol, compound **8**’s anti-HepG2 activity slightly decreased to 37 μM, but its toxic effect on normal liver cells LO2 significantly reduced to 180 μM. Compound **10**, a urushiol Pechmann derivative, was resistant to activity of HepG2, essentially maintaining at 28 μM. However, its toxic effect on normal liver cell LO2 was reduced by 3–4 fold, reaching 180 μM.

In addition, the introduction of pyridine borate on the phenolic hydroxyl group reduced the activity of anti-HepG2 (from 50 to 67 μM). At the same time, the toxic effect on the normal cells LO2 of liver cancer was also weakened (>120 μM). The derivative introduced a molecule of 2-fluoropyridine borate on the phenolic hydroxyl group, but its activity against hepatoma cells decreased dramatically (almost > 200 μM), and the damage to the normal liver cell LO2 also drastically decreased (all > 200 μM).

### 2.3. Inhibition of Cell Migration

**Figure 2:**
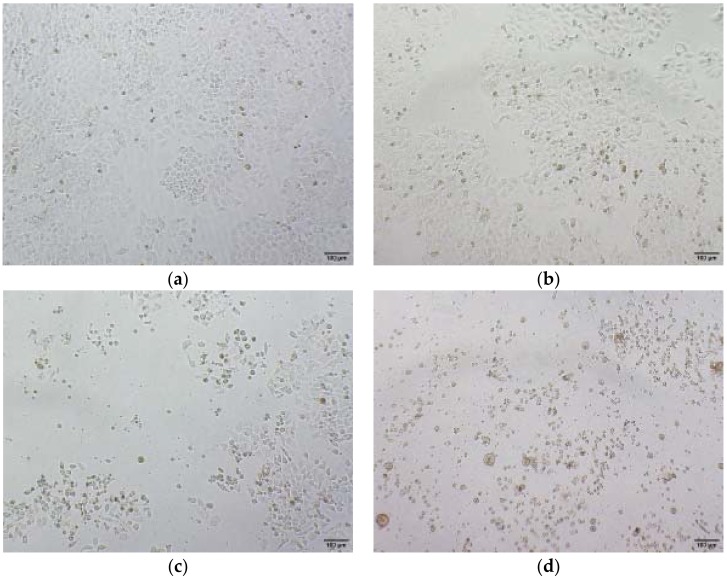
Proliferation of HepG2 cells by compound **1** at different concentrations: (**a**) HepG2 negative control; (**b**) 6.25 μM; (**c**) 25 μM; and (**d**) 50 μM.

As shown in Figure 2, morphological observation showed that proliferation of HepG2 cells was gradually inhibited by addition of the compound **1**. The mortality rate of HepG2 cells was increasing faster on higher concentration of compound **1**. The inhibition of cell proliferation strengthened over time. The negative control group showed uniform cytoplasm, clear nucleoli, full cells and good spindle shape. Besides, with the increase of compound **1**’s concentration, the morphological changes of cells were obvious, and the number of cells decreased significantly. The cells became round, the volume became smaller and the refraction decreased. When the cells were treated with high concentration of compound **1**, the cells were suspended and shed. The cells no longer adhered to the wall. They gradually became round, and the membrane ruptured, shrank and finally lost vitality.

### 2.4. Western Blot Results

**Figure 3:**
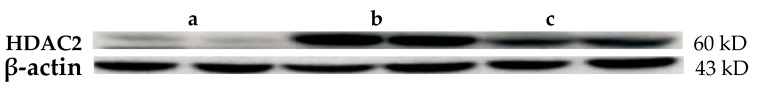
The protein expression of HDAC2 in different groups ((**a**) LO2 cells; (**b**) HepG2 cells; and (**c**) Compound **1** on HepG2 cells).

The total protein was extracted from the logarithmic growth phase of hepatoma HepG2 cell line, and the expression of HDAC2 was detected by Western blot method with urushiol derivative compound **1**(see Figure 3). The Western blot analysis of HDAC2 in HepG2 cells showed clear bands with correct molecular weight. Compared with normal hepatocyte LO2 group, the expression of HDAC2 protein in urushiol derivative compound **1** group was significantly lower than that in hepatoma cell group (*p* < 0.05). The results showed that phenol derivative **1** could down-regulate the expression of hepatoma cells and alleviate the pathological changes of HCC. The mechanism was probably related to the inhibition of HDAC2 expression.

### 2.5. FCM-Flow Cytometry Analysis

Based on the preliminary screening results, HepG2 cells were incubated with various concentrations of compound **1** to investigate the effect of PI Single staining assay for cell cycle, Annexin-V FITC/PI Double staining for apoptosis, JC-1 Staining assay mitochondrial membrane potential, and Calcium content detection (methods details are shown in the Supplementary Materials).

#### 2.5.1. PI Single Staining Assay for Cell Cycle

**Figure 4:**
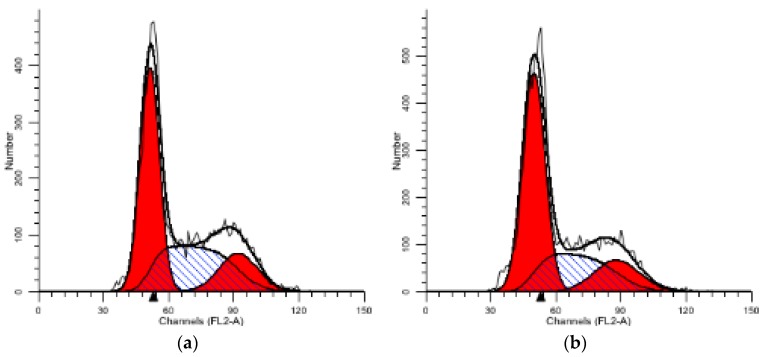

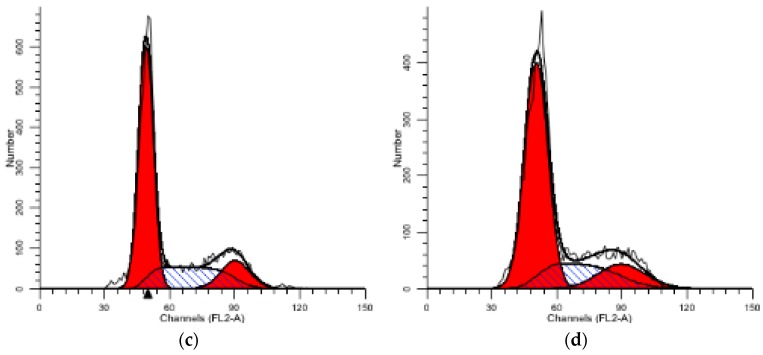
Compound **1** effect on cell cycle of HepG2 cells: (**a**) control group; (**b**) 1 μM; (**c**) 3 μM; and (**d**) 12 μM.

After the cells were tested with three different doses of Compound **1** (1, 3, and 12 μM), they were collected and detected by flow cytometry. HepG2 cells changed significantly in the distribution of cell cycle with the increase of drug concentration. Compared with the blank control group, the proportion of cells in G0/G1 phase of each dose of compound **1** increased gradually, but both the percentage of S stage cells and the proportion of cells in G2/M stage decreased to some extent. Compound **1** could block cells in G0/G1 phase. Compared with blank group, each dose group was significantly different (*p* < 0.05) (see Table 2 and Figure 4).

#### 2.5.2. Annexin-V FITC/PI Double Staining for Apoptosis

**Figure 5:**
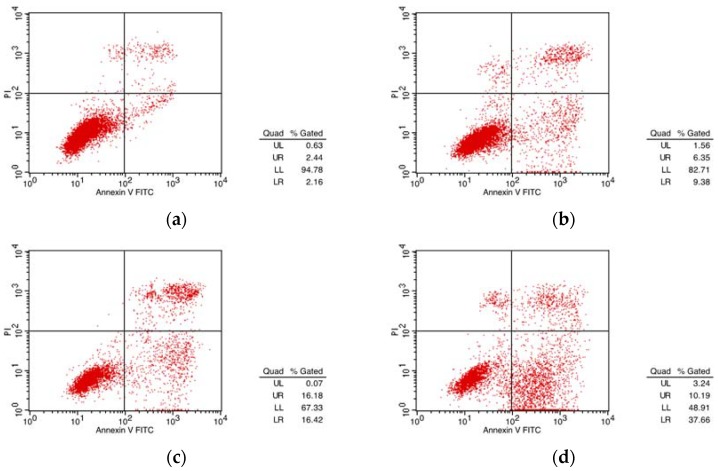
Compound **1** on the apoptosis rate of HepG2 cells: (**a**) control group; (**b**) 1 μM; (**c**) 3 μM; and (**d**) 12 μM.

After treating HepG2 cells with three concentrations of compound **1** for 72 h, flow cytometry was used to detect the apoptosis rate changes. The apoptosis rate of 12 μM group was significantly higher than that of the control group in a dose-dependent manner. The apoptosis rates of the three concentrations were 15.73%, 32.60% and 47.87% (see Table 3 and Figure 5).

#### 2.5.3. JC-1 Staining Assay Mitochondrial Membrane Potential

**Figure 6:**
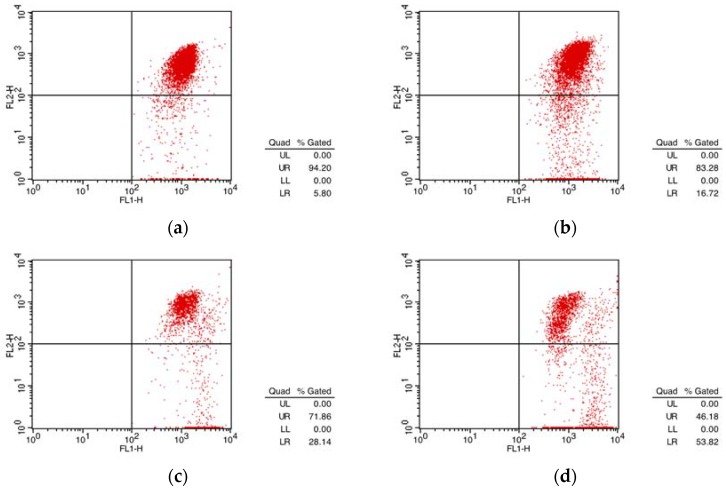
Effect of compound **1** on mitochondrial membrane potential in HepG2 Cells: (**a**) control group; (**b**) 1 μM; (**c**) 3 μM; and (**d**) 12 μM.

As shown in Table 4 and Figure 6, the cells in the control group (UR: 94.20%, LR: 5.8%) and 12 μM experimental group (UR: 46.18%, LR: 53.84%) were stimulated by compound **1**, which reflected the decrease of membrane potential and cell apoptosis.

#### 2.5.4. Calcium Content Detection

**Figure 7:**
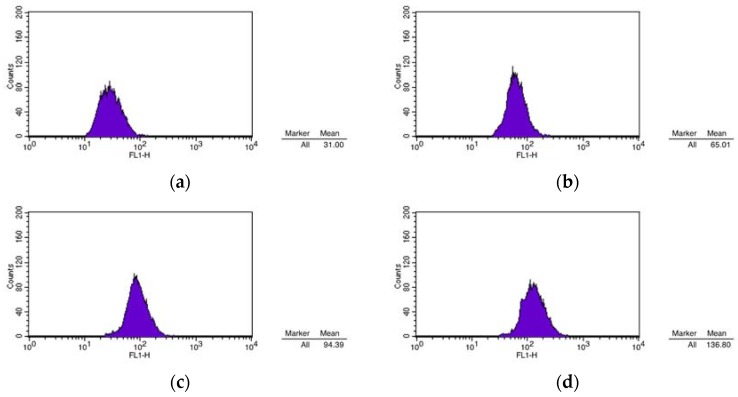
Effect of compound **1** on calcium in HepG2 Cells: (**a**) control group; (**b**) 1 μM; (**c**) 3 μM; and (**d**) 12 μM.

As shown in Table 5 and Figure 7, compared with the blank control group, the cells treated with compound **1** induced apoptosis. They also showed a right-shifted peak of calcium ion concentration, which indicated that the intracellular calcium concentration was increased. This also means that the cell was undergoing apoptosis.

### 2.6. Molecular Docking

Our previous research indicated that the molecular docking technique was used to study the binding mode of urushiol derivatives to HDAC2 receptor [8,9]. The HDAC2 X-ray crystal structure (PDB No. 4LXZ) was downloaded from the RCSB Protein Data Bank (http://www.rcsb.org) with a resolution of 1.85 Å. The UCSF Chimera software [30] established the three-dimensional structure of the ligand and performed energy optimization. Hydrogen atoms were added by using the Dock Prep module, and the AMBER ff14SB force field and AM1-BCC charge were added [31,32]. The molecular surface of the receptor was generated with a 1.4 Å radius probe by using the DMS tool in Chimera. The X-ray crystal structure showed a reasonable binding site for which the sphgen module was used to generate spheres surrounding the active site. A grid file was used to generate a grid file for grid-based energy rating evaluation. Semi-flexible docking was performed by using the DOCK 6.8 program [33,34], which generated 10,000 different conformational orientations and obtained electrostatic and van der Waals interactions between the ligand molecules and the binding sites, and thus the grid score was calculated. By cluster analysis (RMSD threshold of 2.0 Å), the best scoring conformation was obtained. Finally, images were generated by using PyMOL (Schrödinger, LLC. The PyMOL molecular graphics system, version 1.8, 2015).

#### 2.6.1. Conjunction Conformation Score

The DOCK 6.8 program was used to predict the pattern of binding of the compound **1** in HDAC2. The calculation results showed that the binding sites had multiple docking conformations, and the scoring conditions are illustrated in Table 6. According to the scoring and combining mode, we chose the first docking conformation to analyze the combining mode.

#### 2.6.2. Binding Pattern Analysis

**Figure 8:**
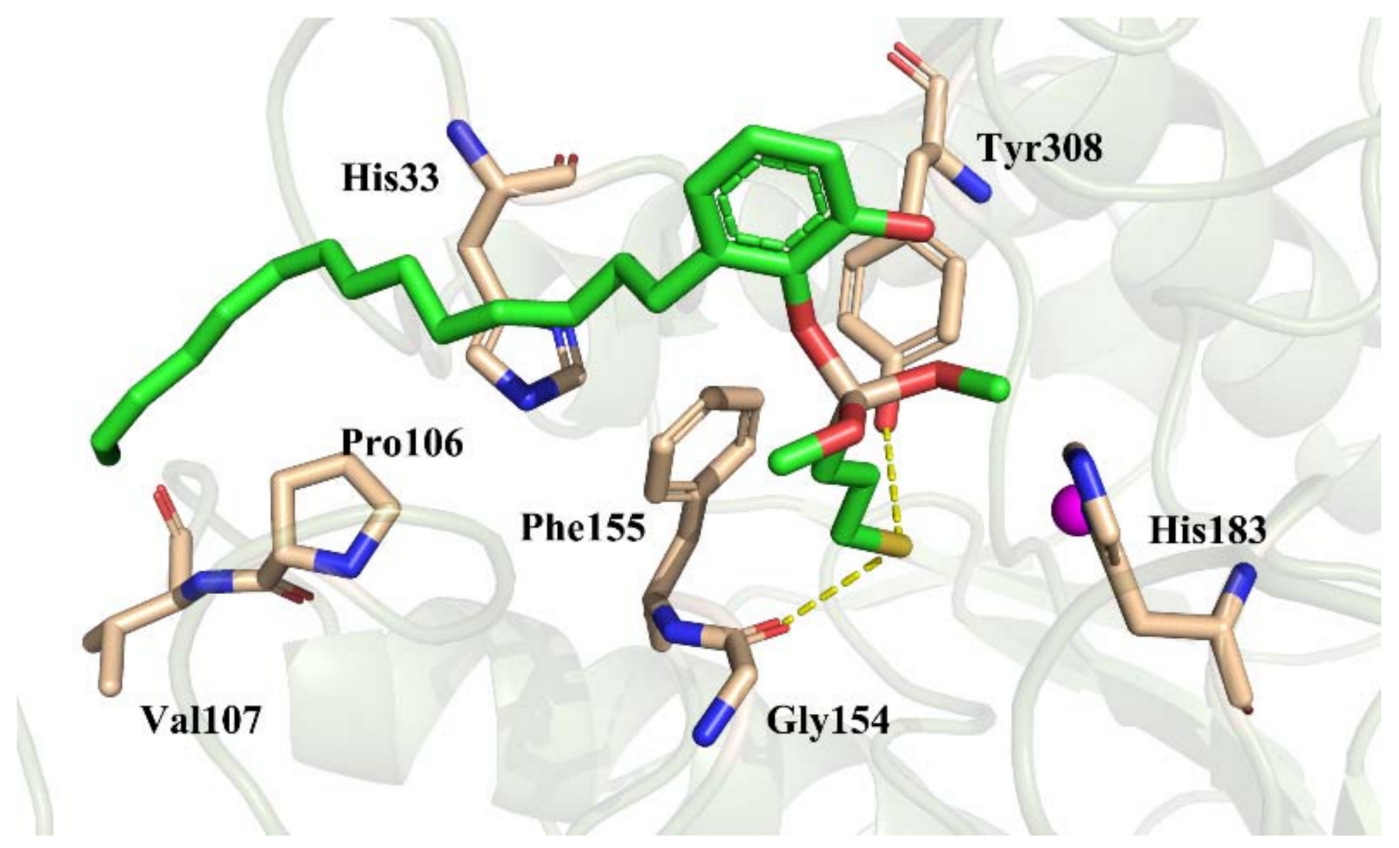
Molecular docking modeling of compound **1** in the active site of HDAC2. In the picture, the ligands (green) and the key residues (wheat) are denoted by sticks, and the protein smudge color by cartoon. A yellow dotted line represents a hydrogen bond.

As shown in Figure 8, thiol sulfur atoms on compound **1** formed hydrogen bonding interactions with Gly154 and Tyr308 respectively, which made compound **1** bind more closely to HDAC2. At the same time, compound **1** also formed hydrophobic interactions with the residues His33, Pro106, Val107, Gly154, Phe155, and His183, and it was provided with a strong van der Waals force by the hydrophobic action.

## 3. Materials and Methods

### 3.1. General

All AR solvents were used without special treatment. All the reactions were monitored by TLC on silica gel F254 plates (Qingdao Haiyang Inc., Qingdao, China) for detection of the spot. Silica-gel column chromatography was performed for purification of the products. MS data were obtained on an Agilent 7890 mass spectrometer. Nuclear magnetic resonance (NMR) spectra were measured on Bruker AV-300 or AV-400 spectrometers. Chemical shifts were expressed in delta (δ) units and coupling constants in Hz. The chemical shifts were reported in parts per million (ppm) and the following abbreviations were used: singlet (s), doublet (d), triplet (t), quartet (q), multiplet (m), and doublet of doublets (dd). The purity of all compounds (urushiol and its derivatives) was determined by HPLC (Shimadzu Co., Tokyo, Japan) and found to be in the 95–99% range.

### 3.2. Plant Material

The lacquer was harvested from Lichuan, Enshi city, Hubei Province, China and identified by our laboratory team. All lacquers were deposited in a vacuum bag for preservation. The crude extract of urushiol was isolated from the lacquer above. Then the C15 triene urushiol was purified by silica gel column chromatography and characterized by spectroscopic and chemical methods. *R*_f_ = 0.81(PE:EA = 1:1).

### 3.3. Extraction and Isolation

At 99.99% N_2_ atmosphere, the raw lacquer (triene urushiol content was more than 50% in the total content of lacquer) was dissolved in MeOH with ultrasonic auxiliary dissolution; the 200~300 mesh gel was used in column, and the mass ratio of silica gel to concentrated enamel was 3–4:1; the eluent was methanol (the volume ratio of raw lacquer quality to methanol was 50:200–400 (g:mL). The eluent were EA and PE, the total volume was 500 mL, and the volume of EA was 10–50 mL. The flow rate of mixed elution was limited between 10 and 15 mL/min, while the EA volume varied from 10 mL to 50 mL. The ratio of EA to PE was 10:490 when the pink ribbon was 10 cm, near to the column sand core. The color change of column silica gel ribbon was a reference for the experiment with the eluent polarity changing from large to small. The chromatography column colors were black-dark, brown-dark, deep yellow, yellow, light red, and light pink (from top to bottom). The first outflow of the pink eluent was collected, and the eluent was concentrated to produce the high purity triene urushiol (85%); then, the column was eluted with a C18 silica gel. PE/EA (4:1) was used as eluent to obtain four saturation urushiols which were later concentrated under reduced pressure to generate a crude residue with more than 95% purity for cryopreserving.

### 3.4. synthesis of Triene Uruhsiol Hydroxyl Protection Compounds

Synthesis of **1**, **2**, and **3** [14]: A solution of triene urushiol (0.3 mmol) and K_2_CO_3_ (1.2 mmol) and propargyl bromide (110 mg, 0.7 mmol) was dissolved in dry acetone (1 mL). The mixture was stirred at 30 °C for 24 h and quenched with H_2_O (30 mL) in an ice bath. The solution was evaporated to remove acetone and extracted with CH_2_Cl_2_ (2 × 30 mL). The organic layer was washed with saturated NaHCO_3_ and brine, dried over MgSO_4_, filtered, and concentrated. The residue was purified by column chromatography with CHCl_3_−MeOH (100:1) to afford compound **3**. The synthesis methods of compounds **1** and **2** followed the path above.

Compound **1**: yield 57%; brown oil; *R*_f_ = 0.90; ^1^H-NMR (400 MHz, CDCl3) δ 7.52 (-OH), 7.29 (s, 1H), 7.00 (s, 1H), 6.71 (s, 1H), 6.36 (t, *J* = 4.36 Hz, 1H), 6.00 (t, *J* = 4.06 Hz, 1H), 5.65 (t, *J* = 9.65 Hz, 1H), 5.46–5.37 (m, 5H), 3.52 (s, 1H), 2.61 (t, *J* = 3.98 Hz, 2H), 0.90 (t, *J* =2.65 Hz, 1H). ^13^C-NMR (101 MHz, CDCl3) δ 143.16, 142.09, 136.86, 132.34, 131.11, 129.97, 129.41, 126.83, 125.57, 124.34, 121.99, 120.01, 112.87, 58.77, 50.83, 27.18, 19.18, 14.12, 13.31. ESIMS *m*/*z* 167.0 [C_5_H_13_O_2_SSiH_2_]^+^.

Compound **2**: yield 45%; brown oil; *R*_f_ = 0.91; ^1^H-NMR (400 MHz, CDCl3) δ 7.65 (m, 1H), 7.45 (m, 1H), 7.28 (s, 1H), 5.31–5.28 (d, *J* = 15.4 Hz, 2H), 1.18 (m, 8H). ^13^C-NMR (101 MHz, CDCl3) δ 167.70, 132.33, 13.71. ESIMS *m*/*z* 479.3 [C_26_H_42_O_4_SSi]^+^.

Compound **3**: yield 67%; brown oil; *R*_f_ = 0.82; ^1^H-NMR (400 MHz, CDCl3) δ 7.52 (-OH), 7.00 (m, 1H), 6.79–6.77 (m, 3H), 6.35 (t, *J* = 4.16 Hz, 1H), 5.98 (t, *J* = 4.46 Hz, 1H), 5.64 (m, 1H), 5.44–5.35 (m, 3H), 4.73 (d, *J* = 2.3 Hz, 2H), 3.73 (d, *J* = 7.0 Hz, 1H). ^13^C-NMR (101 MHz, CDCl3) δ 150.44, 144.44, 143.90, 132.30, 131.10, 129.90, 129.39, 126.78, 125.55, 124.28, 123.40, 119.09, 110.16, 78.32, 76.73, 57.07, 30.62, 29.73, 29.71, 29.63, 29.49, 29.42, 29.25, 27.18. ESIMS *m*/*z* 352.3 [M-H]^+^.

Synthesis of compounds **4**–**7** [19]: A solution of triene urushiol (314 mg, 1 mmol) and phenylboronic acid (1.0 eq) was dissolved in DCM/EA (0.5 mL) and heated to 60 °C for 2 h in a pressure tube. After being concentrated in a vacuum, pure compound **4** (400 mg, 100%) was obtained. Repeat the step above to get compounds **5**, **6**, and **7**, and their yields were 70–80%. The synthesis of compounds **14**, **15**, and **16** were also the same as the synthesis step of compound **4**, and at r.t. for 2 h in a pressure tube.

Compound **4**: yield 75%; brown oil; *R*_f_ = 0.75; ^1^H-NMR (400 MHz, CDCl3) δ 7.42 (d, *J* = 3.06 Hz, 1H), 7.34 (s, 1H), 7.17 (m, 1H), 7.04 (d, *J* = 2.80 Hz, 1H), 6.94 (t, *J* = 3.86 Hz, 1H), 6.87 (d, *J* = 2.85 Hz, 1H), 6.59 (s, 2H), 6.26 (t, *J* = 8.81 Hz, 1H), 6.15 (s, 2H), 5.89 (t, *J* = 2.65 Hz, 1H), 5.34–5.26 (m, 2H). ^13^C-NMR (101 MHz, CDCl3) δ 148.13, 146.81, 145.74, 132.30, 131.08, 131.06, 129.93, 129.41, 129.22, 127.90, 126.85, 125.57, 124.30, 123.18, 122.33, 121.40, 119.24, 114.70, 114.08, 13.29. ESIMS *m*/*z* 449.4 [C_27_H_34_BNO_2_]^+^.

Compound **5**: yield 72%; brown oil; *R*_f_ = 0.74; ^1^H-NMR (400 MHz, CDCl3) δ 9.69 (s, 1H), 7.98 (s, 1H), 7.49 (s, 1H), 7.27, 7.20, 7.17, 6.97 (s, 6H), 5.27, 5.19, 3.39. ^13^C-NMR (101 MHz, CDCl_3_) δ 173.89, 173.44, 148.25, 148.18, 147.66, 130.74, 130.56, 130.26, 130.24, 129.43, 128.65, 128.47, 125.01, 124.55, 119.69, 34.63, 32.49, 30.29, 29.91, 27.75, 25.43, 24.44, 23.26, 14.67.ESIMS *m*/*z* 479.2 [M+C_6_H_7_]^+^. 

Compound **6**: yield 80%; brown oil; *R*_f_ = 0.70; ^1^H-NMR (400 MHz, CDCl3) δ 9.73 (s, 1H), 8.01 (s, 1H), 7.52 (d, *J* = 2.80 Hz, 1H), 7.33 (d, *J* = 1.88 Hz, 1H), 7.30 (d, *J* = 1.50 Hz, 1H), 7.23 (s, 1H), 7.10 (m, 1H), 7.00 (s, 1H), 5.30–5.23 (m, 6H), 4.25 (d, *J* = 8.78 Hz, 8H), 4.11 (m, 2H). ^13^C-NMR (101 MHz, CDCl3) δ 172.32, 171.87, 146.68, 146.61, 146.09, 129.17, 128.99, 128.69, 128.67, 127.85, 127.07, 126.90, 123.44, 122.97, 118.12, 33.05, 30.92, 28.72, 28.33, 26.18, 23.85, 22.87, 21.69, 13.09. ESIMS *m*/*z* 419.3 [M]^+^.

Compound **7**: yield 70%; brown oil; *R*_f_ = 0.79; ^1^H-NMR (400 MHz, CDCl3) δ 8.06 (s, 1H), 7.96 (d, *J* = 0.80 Hz, 1H), 7.52 (m, 1H), 7.36 (t, *J* = 2.80 Hz, 1H), 6.70 (t, *J* = 5.80 Hz, 3H), 5.82(m, 1H), 5.3–5.34 (m, 3H), 4.97 (m, 1H), 1.61 (m, 5H), 1.33, 1.26. ESIMS *m*/*z* 448.1 [M + H]^+^.

Synthesis of compound **8** [15]: Triene urushiol (1.0 mmol), K_2_CO_3_ (3.5 eq.) and 1,3-dibromopropane (1.3 eq.) were added to EtOH (1 mL) in turn, and then the mixture was heated at reflux for 5 h. The resulting mixture was washed with H_2_O (5 mL) and organic layer was concentrated in vacuum to acquire the crude product, which was purified by column chromatography to afford the corresponding compound **8** with PE–EA (4:1, *v*/*v*) as eluents.

Compound **8**: yield 70%; brown oil; *R*_f_ = 0.81; ^1^H-NMR (400 MHz, CDCl3) δ 6.83–6.75 (m, 3H), 6.34 (m, 1H), 6.00 (m, 1H), 5.63 (m, 1H), 5.40–5.35 (m, 3H), 4.18 (m, 2H), 3.58 (t, *J* = 1.80 Hz, 2H), 2.02, 1.74 (t, *J* = 1.10 Hz, 4H). ^13^C-NMR (101 MHz, CDCl3) δ 151.66, 149.70, 126.81, 126.78, 122.70, 122.68, 119.22, 119.20, 77.34, 70.48, 70.47, 27.23, 27.15, 14.10, 13.30. ESIMS *m*/*z* 405.1 [M]^+^.

Synthesis of compound **9** [13]: To a solution of triene urushiol (1.0 mmol) in EA (1 mL) at 0 °C was added TEA (5.06 g, 50.0 mmol) and MsNH_2_ (25 mg), successively. After the addition of MsNH_2_, the mixture was vigorously stirred for 2 h. To the slurry was then added H_2_O (10 mL). The two-phase mixture was separated. The organic layer was washed with H_2_O (25 mL) and dried (MgSO_4_). Removal of solvent under vacuum, the corresponding pure compound **9** was obtained.

Compound **9**: yield 30%; brown oil; *R*_f_ = 0.80; ^1^H-NMR (400 MHz, CDCl3) δ 6.71–6.70 (t, *J* = 4.45 Hz, 3H), 6.34 (t, *J* = 4.80 Hz, 1H), 5.98 (t, *J* = 3.00 Hz, 1H), 5.63 (m, 1H), 5.42–5.35 (m, 3H), 3.71 (s, 3H). ^13^C-NMR (101 MHz, CDCl3) δ 143.08, 141.96, 132.31, 131.07, 129.39, 129.37, 126.83, 125.56, 124.33, 122.06, 120.06, 58.63, 13.30. ESIMS *m*/*z* 436.3 [M + 2Na]^+^.

Syntheses of Compound **10** [16]: In a pressure tube (25 mL), *p*-Toluene (1 eq.) was added into the mixture of triene urushiol (1 mmol) and ethyl acetoacetate (3 eq.), and the reaction mixture was vigorously stirred at ambient temperature for 8 h. The progress of reaction was monitored by TLC (eluent, PE:EA = 4:1). After the completion of the reaction, water (10.0 mL) was added into the reaction mixture. Evaporate the solvent to afford the pure compound **10** in excellent yields, and **10** was regarded as the reagent for compounds **12**–**16**.

Compound **10** (8-hydroxy-4-methyl-7-((8Z,11E,13Z)-pentadeca-8,11,13-trien-1-yl)-2H-chro men -2-one) yield 90%; Yellow liquid; *R*_f_ = 0.83; ^1^H-NMR (400 MHz, CDCl3) δ 6.88–6.48 (m, 5H), 6.49(d, *J* = 5.60 Hz, 1H), 6.22 (s, 1H), 6.02–5.88 (m, 3H), 2.94 (m, 2H), 2.68 (s, 2H),2.43(t, *J* = 0.98 Hz, 4H), 2.11(d, *J* = 7.01 Hz, 2H), 1.94(m, 2H), 1.50 (m, 2H), 1.36 (m, 2H), 1.27 (m, 3H). ^13^C-NMR (101 MHz, CDCl3) δ 167.46, 165.57, 164.44, 161.42, 155.03, 143.34, 142.18, 124.42, 122.44, 120.20, 119.95, 112.86, 112.07, 110.26, 106.90, 50.10, 30.13, 28.45, 28.28, 27.07, 25.15, 21.19, 19.51, 15.53, 14.11, 14.04. ESIMS *m*/*z* 448.4 [M + SiC_3_H_9_]^+^.

Syntheses of Compound **11**: The purified triene urushiol (0.3 mmol) was etherified with methyl iodide (0.6 mmol) in MeCN (1 mL) and the crude product was concentrated under vacuum. The crude product was recrystallized in methanol (saturated urushiol dimethyl ether was not recrystallized) to give pure target compound **11**.

Compound **11**: yield 70%; Yellow liquid; *R*_f_ = 0.81; ^1^H-NMR (400 MHz, CDCl3) δ 6.94–6.65 (m, 3H), 6.34 (t, *J* = 4.08 Hz, 1H), 5.97 (t, *J* = 4.99 Hz, 1H), 5.61–5.35 (m, 3H), 3.93 (s, 3H), 3.79 (s, 3H). ESIMS *m*/*z* 341.0 [M − H]^+^.

Compound **12**: yield 62%; Yellow liquid; *R*_f_ = 0.70; ^1^H-NMR (400 MHz, CDCl_3_) δ 6.73 (m, 1H), 6.71 (s, 1H), 6.67 (m, 1H), 6.23 (s, 1H), 5.98 (s, 1H), 5.89 (s, 1H), 2.39 (t, *J* = 9.80 Hz, 3H), 2.25 (m, 3H), 1.83 (s, 3H). ^13^C-NMR (101 MHz, CDCl_3_) δ 167.30, 161.19, 154.82, 112.88, 21.21, 19.53. ESIMS *m*/*z* 421.3 [M − H]^+^.

Compound **13**: yield 66%; Yellow liquid; *R*_f_ = 0.50; ^1^H-NMR (400 MHz, CDCl3) δ 7.51 (s, 1H), 7.13 (s, 1H), 6.70 (s, 1H), 6.03 (d, *J* = 21.1 Hz, 1H), 5.44–5.17 (m, 6H), 4.64 (s, 2H), 3.40 (s, 1H), 2.60(t, *J* = 1.11 Hz, 2H), 2.40 (m, 2H), 2.24 (s, 3H), 2.05 (d, 3H),1.73 (d, 2H),1.60 (m, 2H),1.33 (m, 2H), 1.26(m, 2H), 0.88 (m, 2H). ^13^C-NMR (101 MHz, CDCl3) δ 171.43, 143.17, 142.05, 132.34, 131.04, 130.02, 129.92, 129.40, 129.37, 126.84, 124.36, 121.99, 120.01, 112.85, 61.77, 60.52, 50.89, 31.98, 31.81, 30.64, 29.79, 29.52, 27.23, 22.68, 21.08, 14.20, 13.32. ESIMS *m*/*z* 432.4 [M + H]^+^.

Compound **14**: yield 28%; Yellow liquid; *R*_f_ = 0.90; ^1^H-NMR (400 MHz, CDCl3) δ 7.53 (d, *J* = 8.5 Hz, 1H), 7.52–7.05 (m, 3H), 6.16 (m, 1H), 5.90 (m, 1H), 5.72 (m, 1H), 5.69–5.64 (m, 3H), 3.67 (s, 1H). ESIMS *m*/*z* 535.4 [M + 2H_2_O]^+^.

Compound **15**: yield 25%; Yellow liquid; *R*_f_ = 0.85; ^1^H-NMR (400 MHz, CDCl3) δ 7.72 (t, *J* = 8.6 Hz, 1H), 7.53 (d, *J* = 5.13 Hz, 1H), 7.36 (s, 1H), 7.07 (s, 1H), 6.70 (m, 8H), 6.10 (m, 1H), 5.84 (m, 1H), 5.60 (m, 1H), 5.40–5.34 (m, 3H), 3.67 (s, 2H), 2.40. ESIMS *m*/*z* 405.2 [M + H]^+^.

Compound **16**: yield 23%; Yellow liquid; *R*_f_ = 0.87; ^1^H-NMR (400 MHz, CDCl3) δ 7.79 (m, 1H), 7.52 (d, *J* = 7.85Hz, 1H), 6.71 (s, 7H), 5.87 (m, 1H), 5.63 (m, 1H), 5.16 (m, 1H), 5.05–4.98 (m, 3H), 3.67 (s, 3H). ESIMS *m*/*z* 421.4 [M + H]^+^.

## 4. Conclusions

We found that urushiol derivatives have a better inhibitory effect on HepG2 of hepatoma cells after screening the broad-spectrum anticancer activity of urushiol derivatives, which inspired us to select hepatoma cell HepG2 as the research object. The result showed that urushiol and its derivatives have a good inhibitory effect on the expression of enzyme HDAC2, and their calculated results were better than that of SAHA [8,9]. The purpose of this paper is to explain that urushiol derivative **1** had inhibitory effect on HDAC2, but the specific inhibitory effect of urushiol on enzymes, such as HDACs, needs more extensive research. The main significance of this paper is to synthesize urushiol derivatives for the first time, which is beneficial for inhibiting the expression of HDAC2.

In this paper, the structure–activity relationship showed that compound **1** with the thiol-containing long-chain alkane, compound **4** with the phenylboronic acid and compound **9** with the amino sulfoxide played an effective role in attenuating and increasing the efficiency; the same condition also applied to the urushiol Pechmann derivative. The finding provided the experimental basis for the subsequent synthesis of novel urushiol derivatives with reduced toxicity and enahnced efficacy.

The Grid docking studies revealed that introducing alkane thiol substituents in side chain could increase binding affinity significantly. Molecular docking studies revealed that Zn^2+^ coordination, hydrogen bonding, and hydrophobic interaction contributed to the high binding affinity of these compounds toward HDAC2. Gly154, Tyr308, His33, Pro106, Val107, Gly154, Phe155, and His183 contributed favorably to the binding between enzyme and the compounds. In addition, the results of binding energy decomposition clearly showed that van der Waals and electrostatics interactions provided major contributions to the stability of these complexes. This study showed the promising potential of urushiol and urushiol derivatives as potent HDAC2 binding agents. Further studies on the synthesis and determination of their HDAC2 inhibitory properties are planned.

## Figures and Tables

**Table 1:** Urushiol derivatives inhibitory activity against HepG2 and LO2 in vitro (IC_50_, μM, 72 h).

**Compound**	**1**	**2**	**3**	**4**	**5**	**6**	**7**	**8**
HepG2 ^a^/IC_50_	7.88	197.94	>200 ^b^	15.33	180	>200	89.66	37.25
LO2/IC_50_	150.59	>200	>200	180.78	>200	>200	>200	180.51
**Compound**	**9**	**10**	**11**	**12**	**13**	**14**	**15**	**16**
HepG2 ^a^/IC_50_	15.01	28.75	>200	50.57	65.43	78.66	67.04	150.62
LO2/IC_50_	120.81	198.36	>200	120.54	150.84	>200	>200	>200

^a^ HepG2: human liver hepatocellular carcinoma cells; ^b^ No inhibitory activity at 200 μM.

**Table 2:** Compound **1** effect on cell cycle of HepG2 cells (x ± s, *n* = 7).

Compound	Group/μM	G1 (%)	S (%)	G2 (%)
**1**	CON	48.06	35.8	16.14
	1	54.48	29.72	15.79
	3	60.17	26.19	13.64
	12	65.80	20.40	13.79

*p* < 0.01, compared with control group.

**Table 3:** Effect of compound **1** on the apoptosis rate of HepG2 cells.

Compound	Group	UL (%)	UR (%)	LL (%)	LR (%)	Apoptosis (%)
**1**	CON	0.63	2.44	94.78	2.16	4.6
	1 μm	1.56	6.35	82.71	9.38	15.73
	3 μm	0.07	16.18	67.33	16.42	32.60
	12 μm	3.24	10.19	48.91	37.68	47.87

**Table 4:** Effect of compound **1** on mitochondrial membrane potential in HepG2 Cells.

Compound	Group	Green Fluorescence (%)
**1**	CON	5.80
	1 μm	16.72
	3 μm	28.14
	12 μm	53.82

**Table 5:** Effect of compound **1** on calcium in HepG2 Cells.

Compound	Group	Mean
**1**	CON	31.00
	1 μm	65.01
	3 μm	94.39
	12 μm	136.80

**Table 6:** Docking of ligand with receptor HDAC2 (unit: kcal/mol).

Compound	Pose	Grid Score	Grid_vdw	Grid_es	Int_energy
Ligand 1	1	−54.809010	−47.656796	−7.152214	13.308063
	2	−53.249386	−46.243793	−7.005592	15.280022

## Supplementary Materials

The following are available online. Detection of cell proliferation by MTT, HDAC2 expression, Flow cytometry experimental process and compounds MS; 1H/13C NMR spectrograms.
