# Supplementary material for: Synthesis and Evaluation of C15 Triene Urushiol Derivatives as Potential Anticancer Agents and HDAC2 Inhibitor
Source: Molecules. 2018 May 3;23(5):1074. doi: 10.3390/molecules23051074 (PMC6102549; doi:10.3390/molecules23051074)

Supplementary material

Synthesis and Evaluation of C15 Triene Urushiol Derivatives as Potential Anticancer Agents and HDAC2 Inhibitor

Zhiwen Qi [1,2,3], Chengzhang Wang [1,2,*] and Jianxin Jiang [3]

[1] Institute of Chemical Industry of Forest Products, CAF, Nanjing 210042, China; angelkissgod@163.com
[2] Key and Open Laboratory on Forest Chemical Engineering, SFA, Nanjing 210042, China
[3] College of Material Science and Technology, Beijing Forestry University, Beijing 100083, China; jiangjx@bjfu.edu.cn
* Correspondence: wangczlhs@sina.com; Tel.: +86-139-5194-5535

MS triene urushiol......7
MS Compound 1 T5-3 7
MS Compound 2 T5-5 8
MS Compound 3 F2-1......8
MS Compound 4 X3-6 9
MS Compound 5 X3-5 9
MS Compound 6 X3-5 10
MS Compound 7 N5-1......10
MS Compound 8 L5-1......11
MS Compound 9 M5-3 11
MS Compound 10 N2-1......12
MS Compound 11 N2-1......12
MS Compound 12 N2-1......13
MS Compound 13 S5-1......13
MS Compound 14 S5-2......14
MS Compound 15 S5-3......14
MS Compound 16 S5-3......15
NMR 1H/13C Compound 1 T5-3 16
NMR 1H/13C Compound 2 T5-5 17
NMR 1H/13C Compound 3 F2-1......18
NMR 1H/13C Compound 4 X3-6 19
NMR 1H/13C Compound 5 X3-3 20
NMR 1H/13C Compound 6 X3-5 21
NMR 1H Compound 7 N5-1......22
NMR 1H/13C Compound 8 L5-1......23
NMR 1H/13C Compound 9 M5-3 24
NMR 1H/13C Compound 10 N2-1......25
NMR 1H Compound 11 T5-6 26
NMR 1H/13C Compound 12 N2-1......27
NMR 1H/13C Compound 13 S5-1......28
NMR 1H Compound 14 S5-2 29
NMR 1H Compound 15 S5-3 30
NMR 1H Compound 16 S5-4 31

Detection of cell proliferation by MTT

MTT assay was used to determine the inhibitory effect of the sample on the proliferation of HepG2 cells. The tumor cells of logarithmic growth stage were inoculated with 3.5 * 103 / well in 96 well culture plate. 0.2 ml/pore system was cultured for 72 h with different concentrations of samples. Then 20μL(final concentration 0.5 mg / mL) of MTT were added to each pore for 4 h. The supernatant was carefully adsorbed, DMSO 0.15 mL was added to each pore, and oscillatory mixing was carried out. The absorbance (A) value at 490 nm wavelength was measured by enzyme labeling instrument. The inhibition rate of cell proliferation was calculated as follows: [(A value of negative control group-A value of blank group)-(A value of experimental group A value of blank group)] / (negative pair group A-A value of blank group) × 100.Statistical method. The statistical analysis was carried out by SPSS 17.0 statistical software. The measured data were described by x ±s, and the mean of the two samples was compared with t test ($P < 0.05$).

HDAC2 expression

Materials and methods: Main reagents and instruments

HDAC2, with a molecular weight of 60kD (1 KD=0.9921 Ku), was purchased from Santa Cruz Company. ECL membrane, and Polyacrylamide gel were purchased from AmershamLife Science Company. ECL membrane elution buffer were purchased from PIERCE Company. The cell culture medium and its additive are purchased from Gibco Company. Full-range rainbow Markers, a full molecular weight protein standard, was purchased from Amersham Biosciences. The 60 kD HDAC2 standard was diluted with buffer solution and 1% SDS at 2 μg/ml, then stored at -20℃. FACS Calibur flow cytometry was produced by BD Company in USA. Cell culture 10% SDS- polyacrylamide gel was prepared. The samples were sampled with 20 μL of each sample, 10μL of HDAC2 standard sample with 60kD, and 10μL of 100 V voltage electrophoresis with standard protein sample of full molecular weight. The eggs on the gel were electrophorezed for 1.5 hours. The white matter was transferred to ECL film for 100V, 1h, blocked with PBST containing 5% skim milk powder (PBS solution containing 0.5% Tween-20) at room temperature for 2 hours, then diluted with PBST containing 2% skim milk powder for 2 hours. Compounds 1, 1: 100, 1: 200, 1: 500, 4℃ incubated overnight with 100r/min. PBST solution was washed for 3 times, then reacted with horseradish peroxidase labeled goat anti-rat Ig 1: 7500) for 1.5h at room temperature. The chemiluminescence kit developed and exposed to detect the expression of HDAC2.The used ECL membrane was eluted at room temperature with eluant for 15 min, then stained with Actin antibody 1: 10000) and detected.

Flow cytometry Material

The HepG2 cells were cultured in a saturated humidity incubator with 90% DMEM+10%FBS, 90%MEM+10%FBS, 90%RPMI1640+10%FBS, and HG2A2780, provided by Jiangsu Kaiji Biotechnology Co., Ltd. Main reagents and consumables Cell culture bottle

(FALCON 353014)Penicillin/streptomycin solution) (China Jiangsu Kaiji Biotechnology Co., Ltd.) 0.25% Tripsin-EDTA (China Jiangsu Kaiji Biotechnology Co., Ltd.) KGY001BSs (China Jiangsu Kaiji Biotechnology Co., Ltd. United States GIBCO 31800-105 DMEM (GIBCO 11965-084) (US GIBCO 12571-063)FBS (Gibco 10082147 wellcell culture plate Corning Incorporated 3516)MTT cell proliferation and cytotoxicity test kit (KGA311, Jiangsu Kaiji Biotechnology Development Co., Ltd., China)Cell cycle detection kit (KGA512, Jiangsu Kaiji Biotechnology Development Co., Ltd.)Apoptotic mitochondrial membrane potential detection kit JC-1 (KGA604, Jiangsu Kaiji Biotechnology Development Co., Ltd., China)Annexin V-FITC/PI double staining cell apoptosis detection kit (KGA108, Kaiji Biotechnology Development Co., Ltd., Jiangsu, China)Ca2 GPCR analysis-calcium ion indicator probe Fluo-3, AM (KGAF023)

Method: cell culture

Cell resuscitation

1.1 to mix the warm water from 37 to 40℃, remove the cryopreservation tube from the liquid nitrogen, and immediately plunge into the warm water from 37℃ to 40℃, until the cryopreservation liquid is completely dissolved.

1.2 cell cryopreservation suspension is transferred to a centrifuge tube, add about 5ml medium, and gently mix;

1.3 cell suspension by 800 ~ 1000r/min centrifugal 5min, abandoned above;

1.4 to join the cell pellet culture medium, and gently mix, to transfer the cell suspension to culture flask, make up the medium.

Cell passage

2.1 When the cell coverage in the culture bottle reached 80%-90%, the original medium was sucked out.

2.2 with appropriate trypsin (0.25%) and digestion for 1-2 minutes.

2.3 cells became round, the digestion was terminated with the medium of equal volume of serum containing serum.

2.4 with pipette percussion cells, the cells are suspended, and then were sucked into 15ml centrifuge tube and centrifuged for 5 minutes;

2.5 the supernatant was dropped and the 1-2ml medium was added, and the cells were suspended and transferred to the culture bottle to continue to be cultured.

3 cell cryopreservation

3.1 the suitable trypsin digestion cells were added, and the cell suspension was collected into the centrifuge tube. 1000rpm was centrifuged for 5 minutes, and the supernatant was abandoned.

3.2 to join the cell sediment cryoprotectant, lightly mixed mixing, the cell density of 1 * 106~1 * 107 /ml;

3.3 according to the amount of 1 ~ 1.5ml per tube in the cryopreservation tube, tighten the cap and mark on the refreezing tube, including the cell code and the freezing date.

3.4 in the following order: cooling at room temperature to 4℃(20 minutes) to the freezer (30 minutes), low temperature refrigerator (30℃ for 1 hours), gaseous nitrogen (30 minutes), the liquid nitrogen.

Cell proliferation measured by MTT

Experimental methods:

1) the cells were digested and counted, the cell suspension was prepared by 3 x 104 /ml, and 100 micron L cell suspension was added to each hole in the 96 hole cell culture plate.

2) 96 hole cell culture plate was placed in 37, 5%, CO2 culture box for 24 hours.

3) use the medium to dilute the drug to the desired working fluid concentration, add 100 micron l per hole to the corresponding drug medium, and establish a negative control group and a positive control group (paclitaxel, 20ug/ml).

4) 96 hole cell culture plate was placed at 37℃ and 5% CO2 incubator for 72 hours.

5) 96 hole board for MTT staining, lambda =490nm, OD value determination;

a. 20 μ L MTT (5mg/ml) per hole was added to the culture box for 4 hours.

b. discarding the supernatant, adding 150 μ L DMSO for each hole, and gently mixing the bed for 10 minutes.

c. lambda =490nm, ELISA od readout per hole, the inhibition rate was calculated.

6) calculate the inhibition rate of each group.The inhibition rate (%) = (negative control group OD value of experimental group / OD) negative control group OD x 100%

Detection of cell cycle by PI single staining

1, the logarithmic growth phase were digested and inoculated into six well plates, the next day, after the cells adhered, according to the group set to join the corresponding drug containing medium, and the establishment of the negative control group;

2, after the action of 72h, the cells were digested with 0.25% pancreatin (not containing EDTA).

3, 5 x 105 cells were collected by PBS scrubbing cells (centrifuge 2000rpm, 5min).

4, the single cell suspension prepared was fixed for 2 hours (or overnight) with a volume fraction of 70% ethanol, and stored at 4℃. Before washing, PBS was used to remove the fixative solution (if needed, cell suspension was filtered through 200 mesh mesh).

5 plus 100 mu L RNase A 37℃ water bath 30min;

6, adding 400 L PI dyeing and avoiding light 30min at 4℃.

7 check the machine and record the red fluorescence at the excitation wavelength of 488nm.

Detection of apoptosis by Annexin-V FITC/PI double staining

(1) inoculate the logarithmic growth cells into the six-hole plate. The next day, after the cells are adhered to the wall, according to the group. The culture medium containing medicine was added and the negative control group was set up at the same time

(2) after 72 hours of treatment, the cells were digested with 0.25% trypsin (excluding EDTA).

(3) 5 × 10 ~ 5 cells were collected by PBS washing cells twice (centrifugation 2000rpm for 5

min);

(4) adding 500 μ L Binding Buffer suspension cells;

(5) After adding 5 μ L Annexin V-FITC, adding 5 μ L Pi, mixing;

(6) at room temperature, no light, the reaction time was 5 ~ 15 min, and the apoptosis was detected by flow cytometry.

Detection of mitochondrial membrane potential by JC-1 staining

1inoculate the logarithmic growth cell digestion into the six-hole plate, the next day, after the cells adhered to the wall, according to the group set up to add the corresponding drug medium, and set up a negative control group at the same time;

(2) the cell concentration was collected and adjusted to 1 × 10 6 / ml by PBS washing (centrifugation 2000rpm for 5 min);

(3) 100 μ L 10 × Incubation Buffer and 900 μ L sterilized deionized water were diluted to 1 × Incubation buffer, and then preheated to 37℃;

(4) 500 μ L 1 × Incubation buffer was absorbed and 1 μ L JC-1 was added to prepare JC-1 working fluid.

(5) the cells were incubated in a incubator containing CO 2 at 37 ℃ for 15 ~ 20 min with 500 μ L JC-1 solution.

(6) the cells were collected by centrifugation at room temperature for 5 min and washed twice with 1 × Incubation Buffer.

(7) resuspension cells of 500 μ L 1 × Incubation Buffer;

(8) on the computer test.

Calcium content detection

(1) inoculate the logarithmic growth cells into the six-hole plate. The next day, after the cells are adhered to the wall, according to the group. The culture medium containing medicine was added and the negative control group was set up at the same time.

(2) after 72 hours of treatment, the cells were digested with 0.25% trypsin (excluding EDTA).

(3) 5 × 10 ~ 5 cells were collected by PBS washing cells twice (centrifugation 2000rpm for 5 min);

(4) adding 5 μ M Fluo-3 probe and mixing it to 37 degrees to avoid light for 30 min;

(5) Computer testing

MS triene urushiol

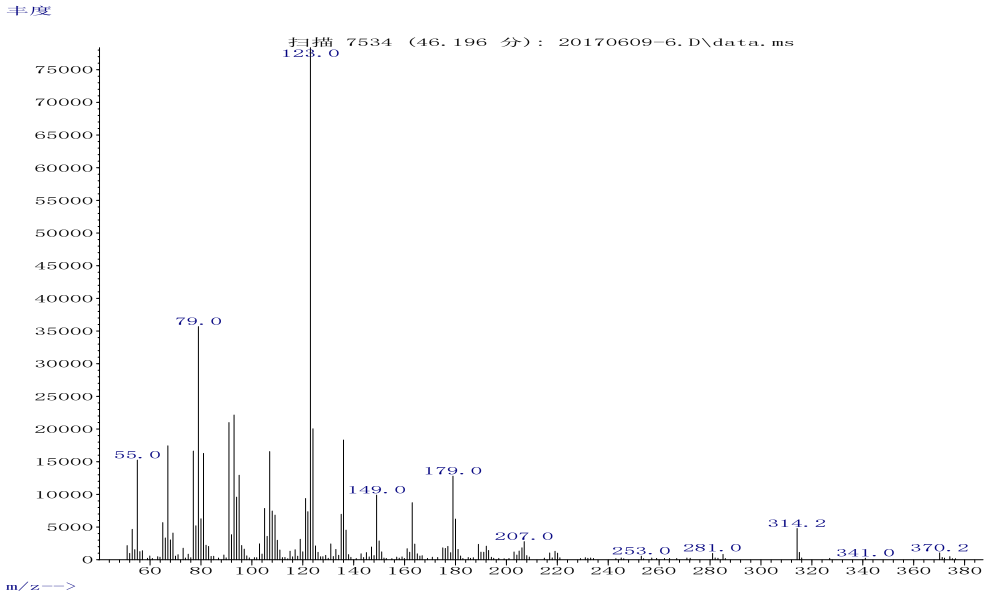

MS Compound 1 T5-3

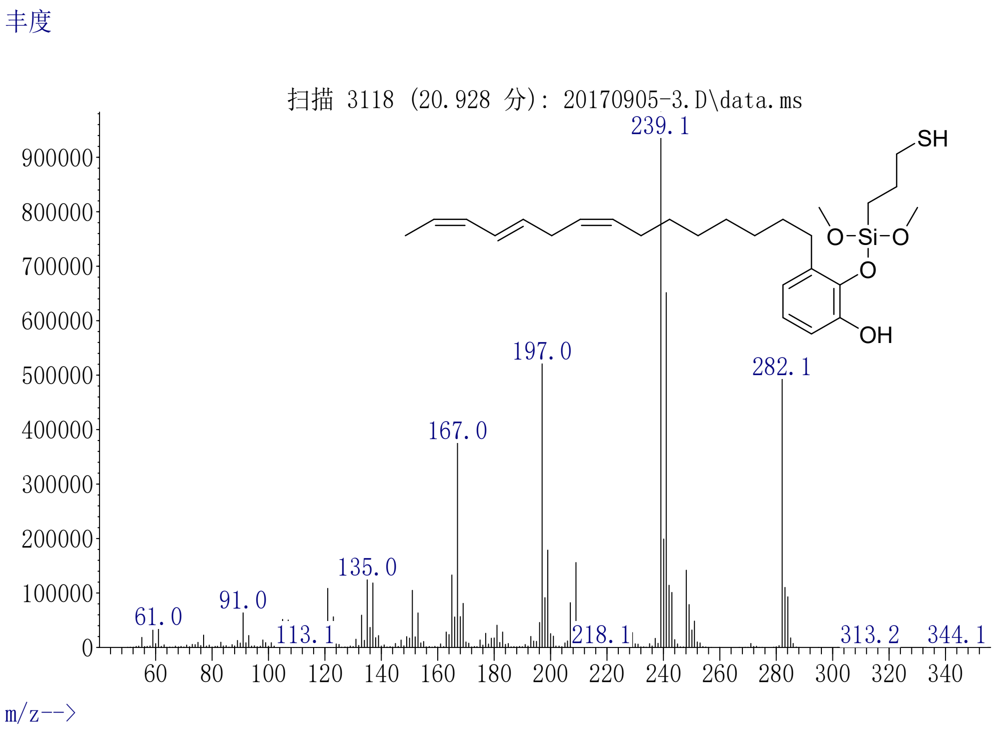

MS Compound **2 T5-5**

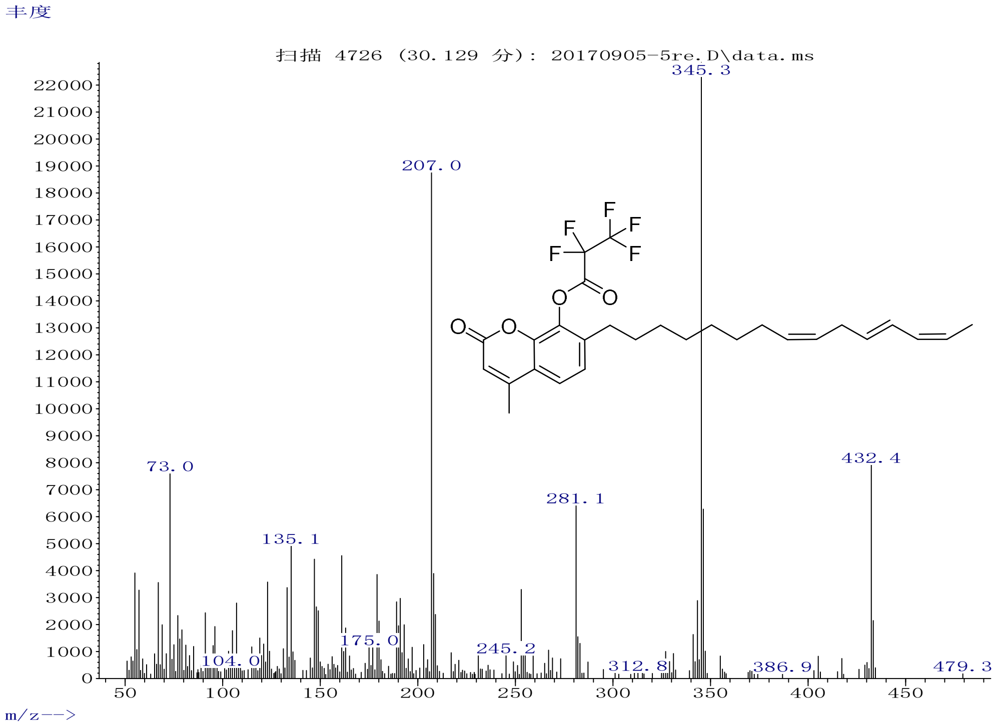

MS **Compound** 3 F2-1

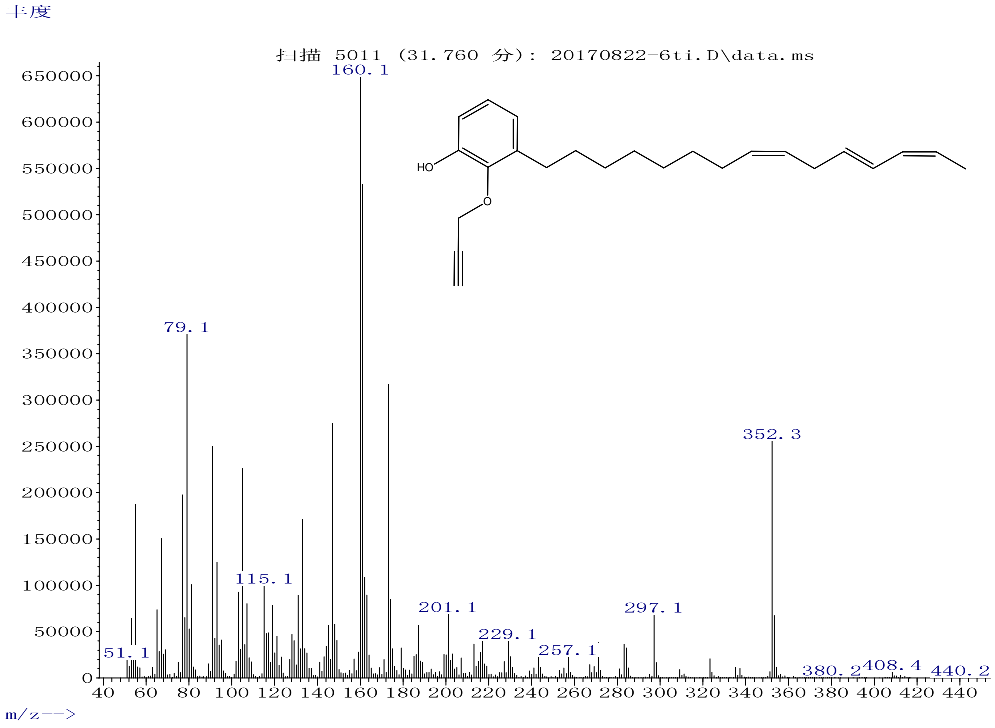

MS Compound 4 X3-6

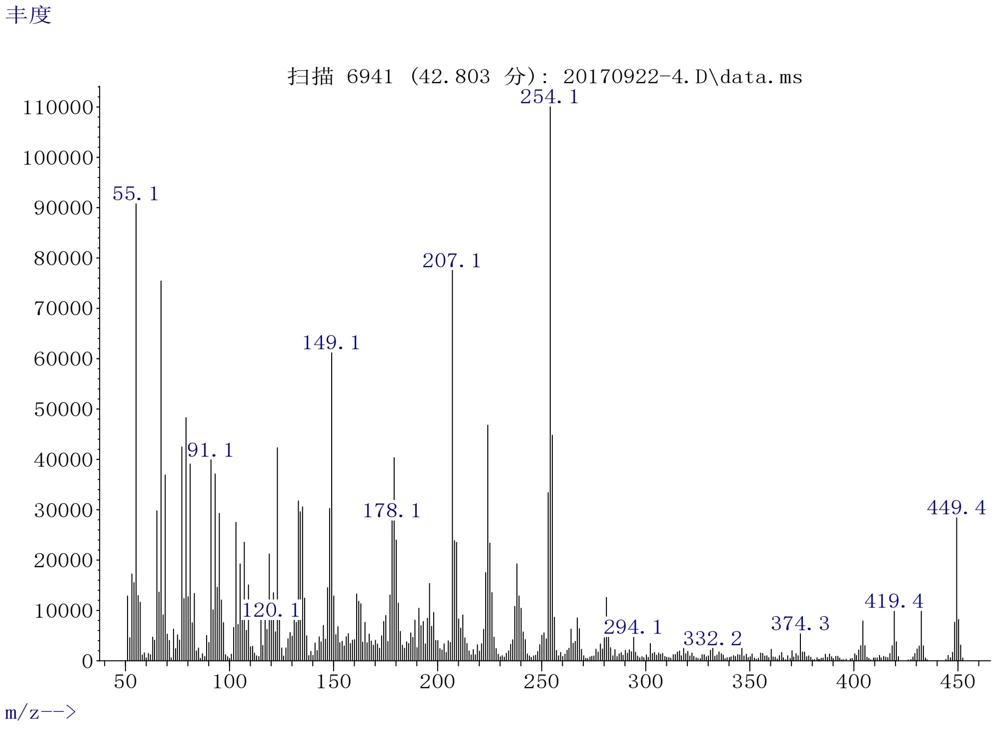

MS Compound 5 X3-5

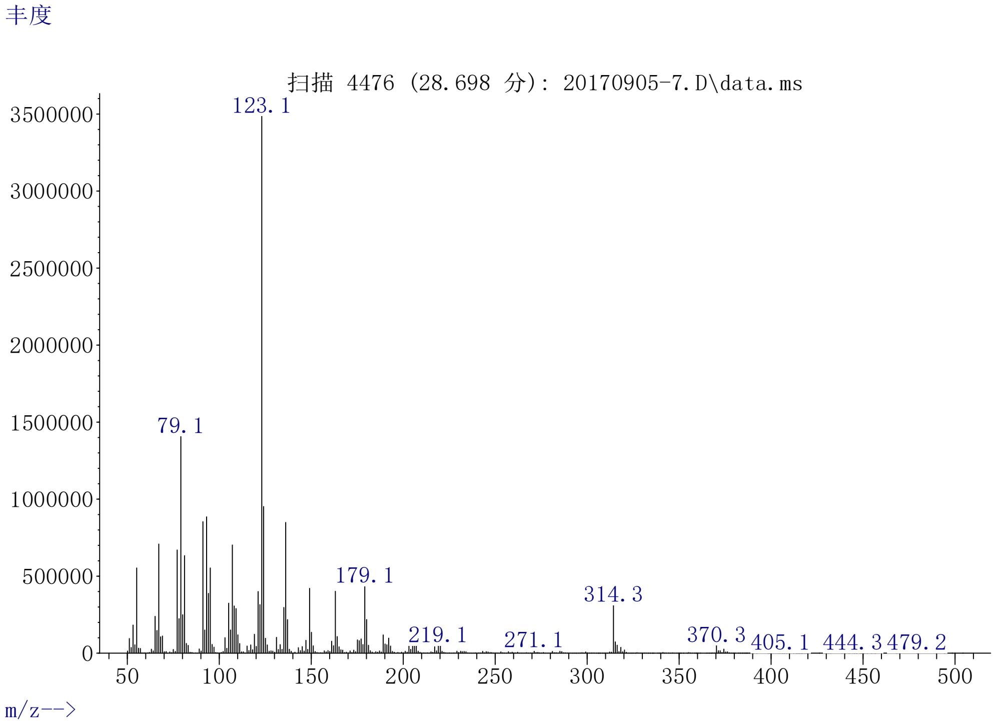

MS Compound 6 X3-5

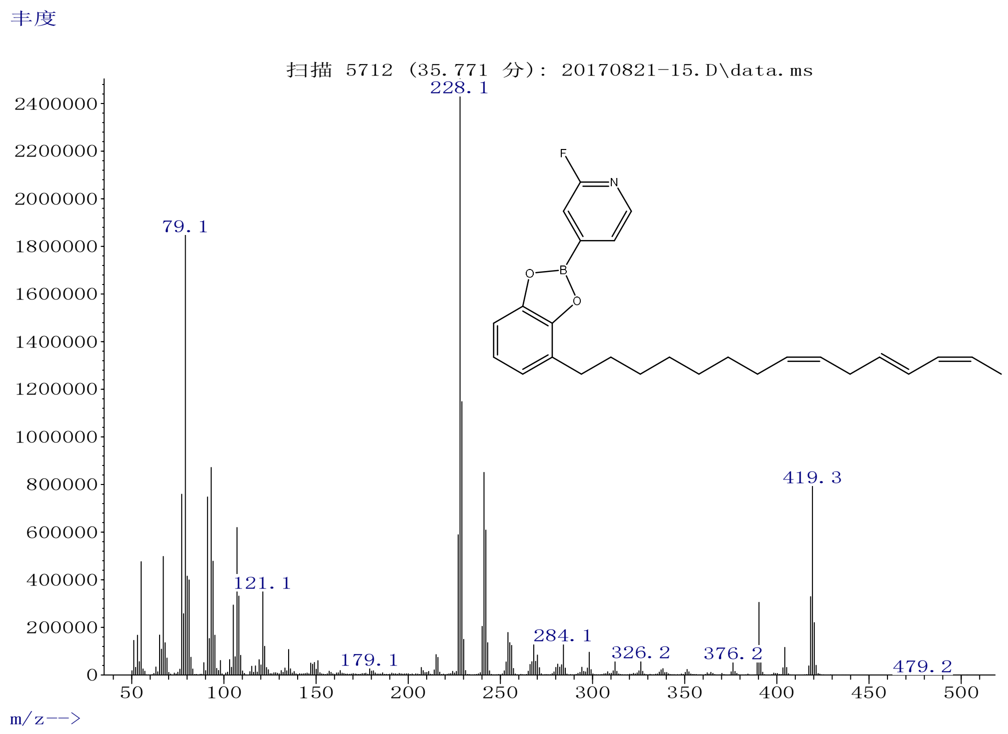

MS Compound 7 N5-1

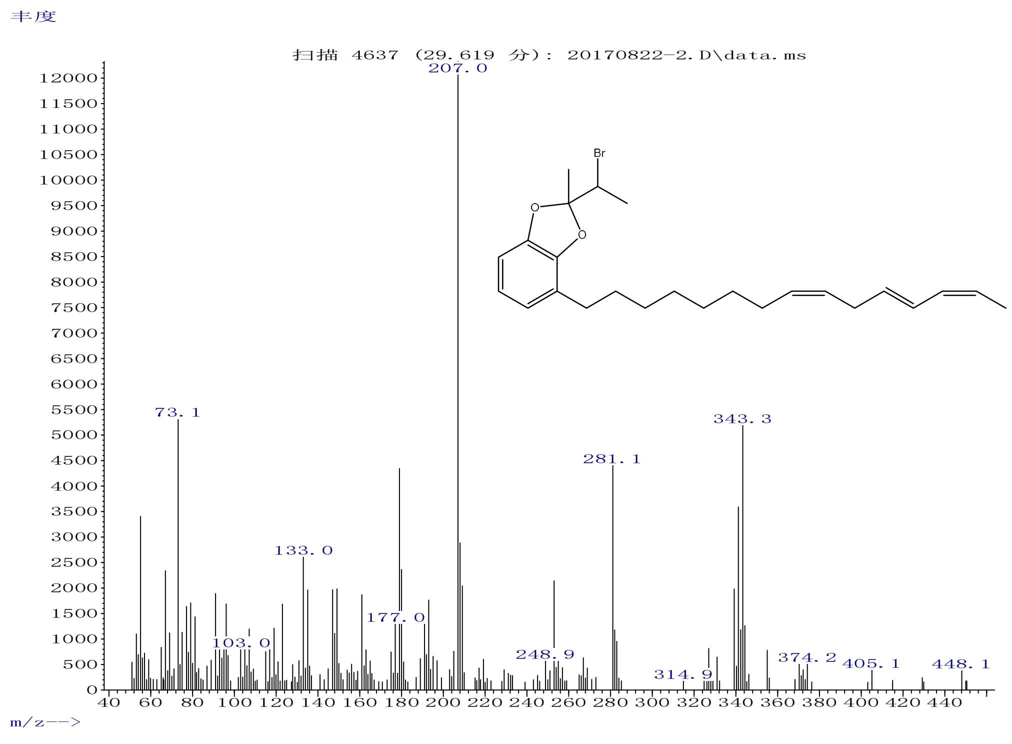

MS Compound 8 L5-1

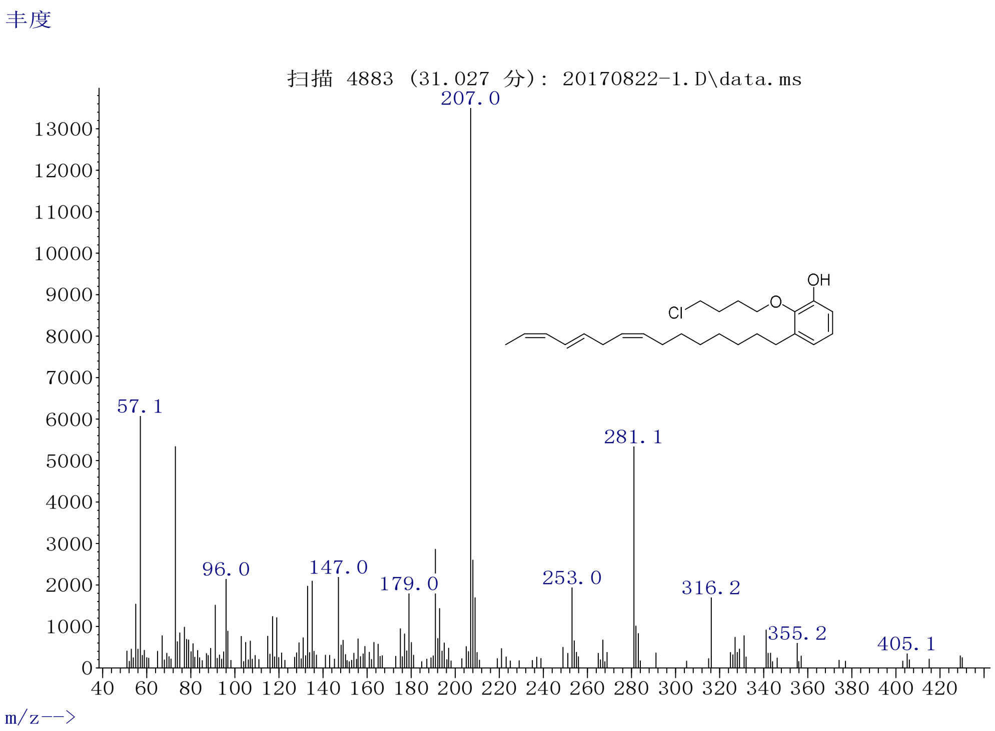

MS Compound 9 M5-3

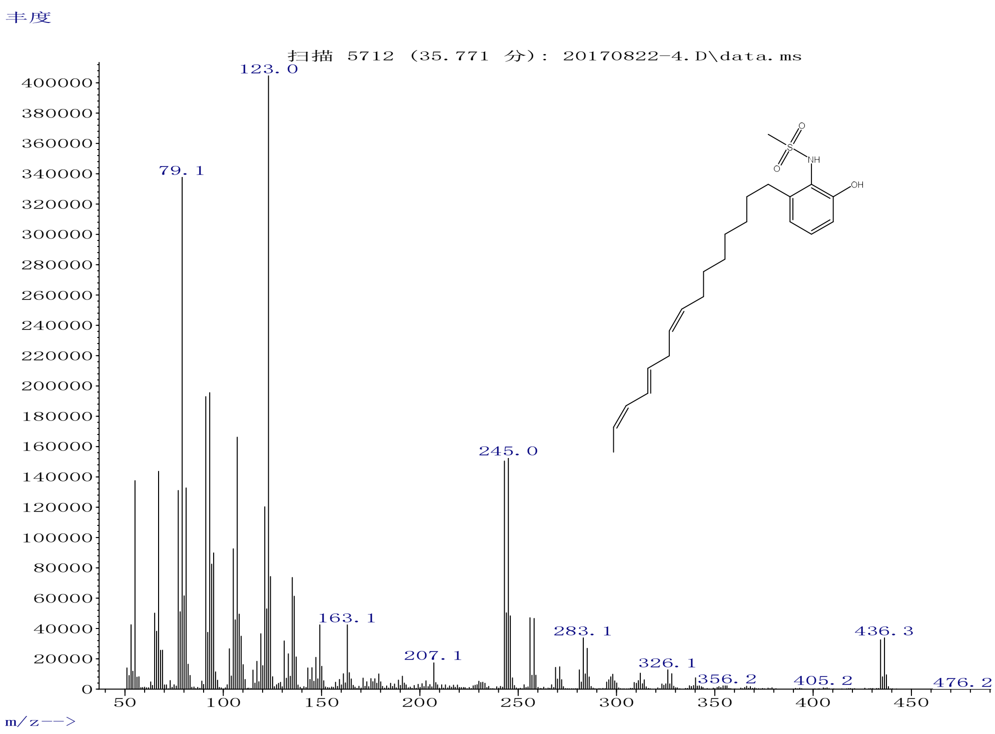

MS Compound 10 N2-1

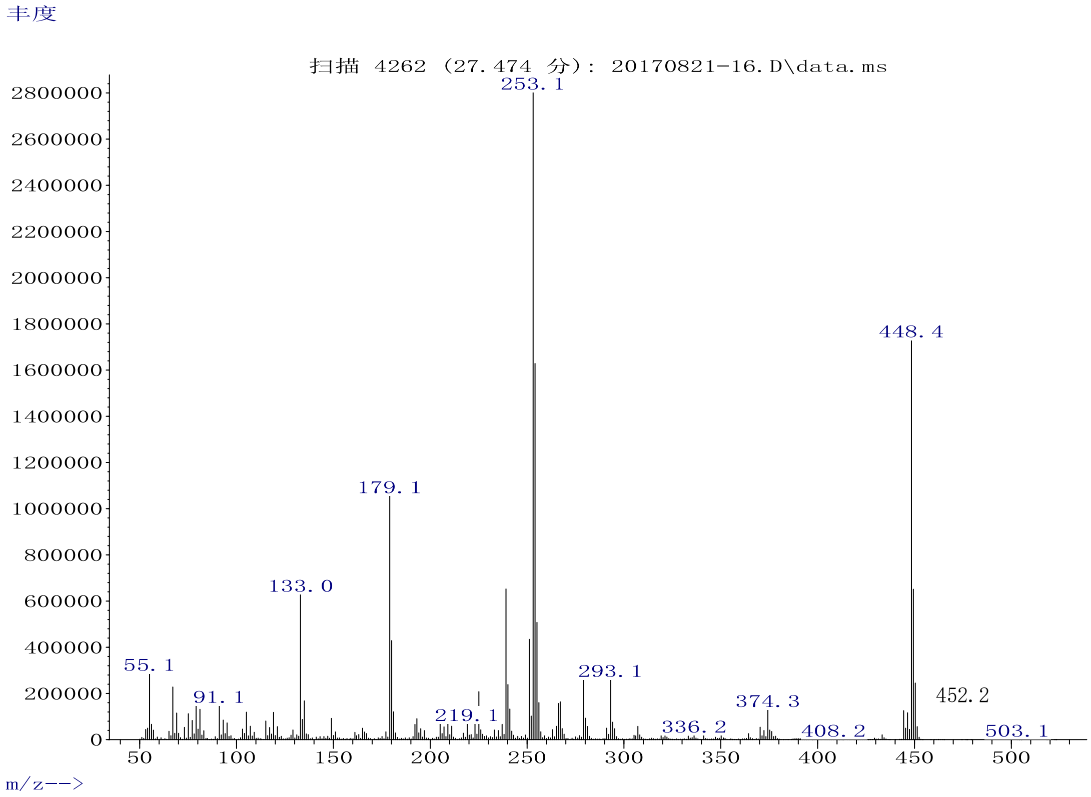

MS Compound 11 N2-1

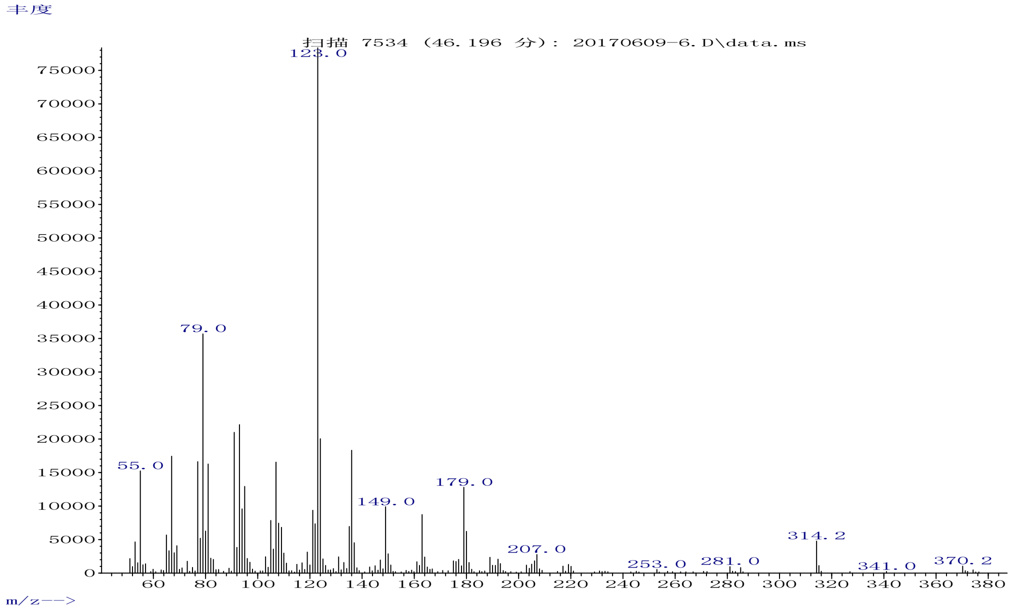

MS Compound 12 N2-1

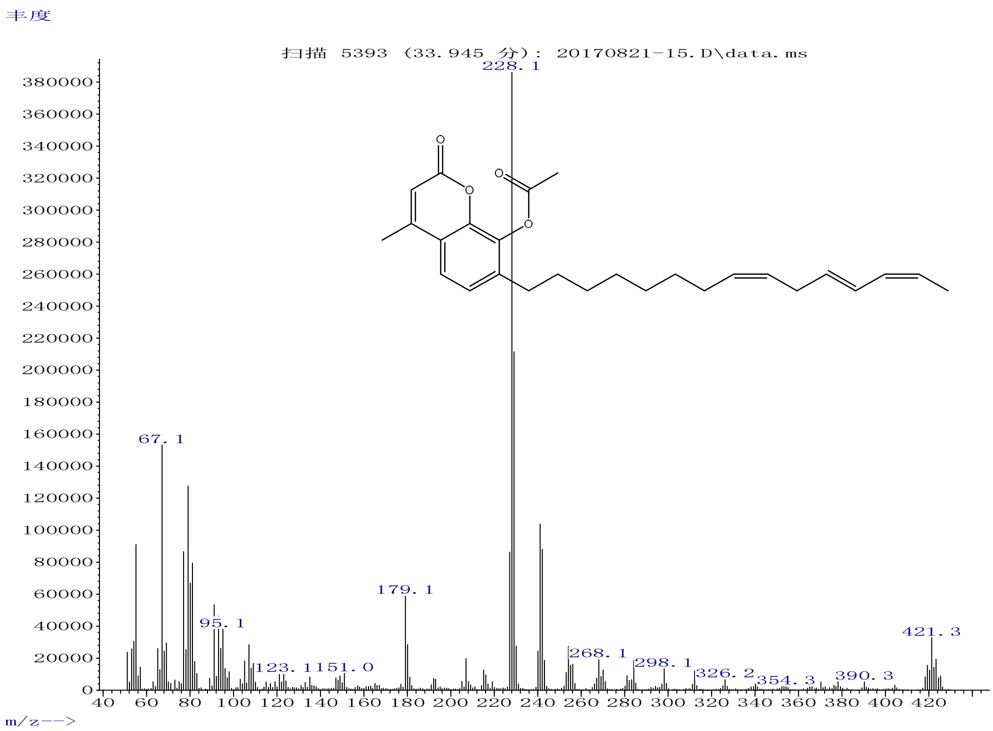

MS Compound **13** S5-1

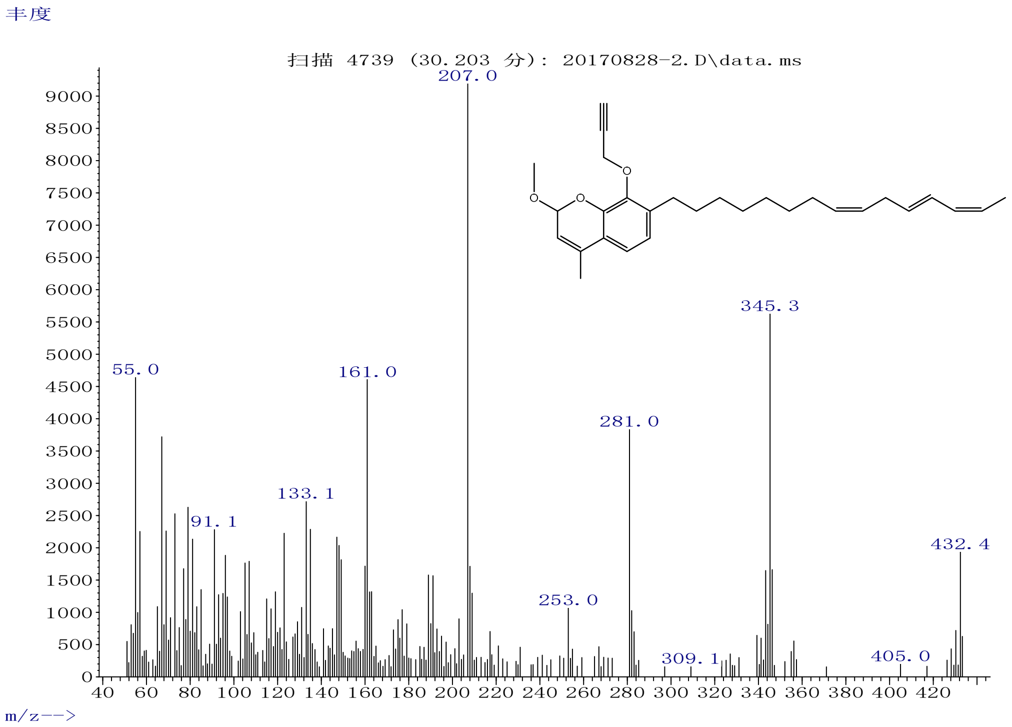

MS Compound 14 S5-2

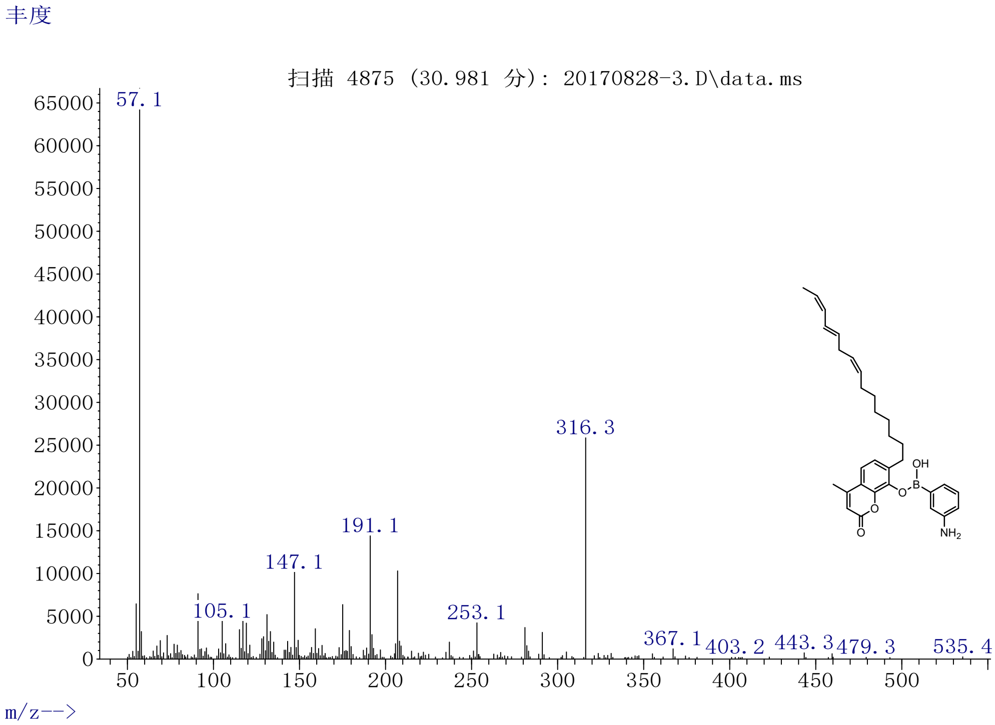

MS Compound 15 S5-3

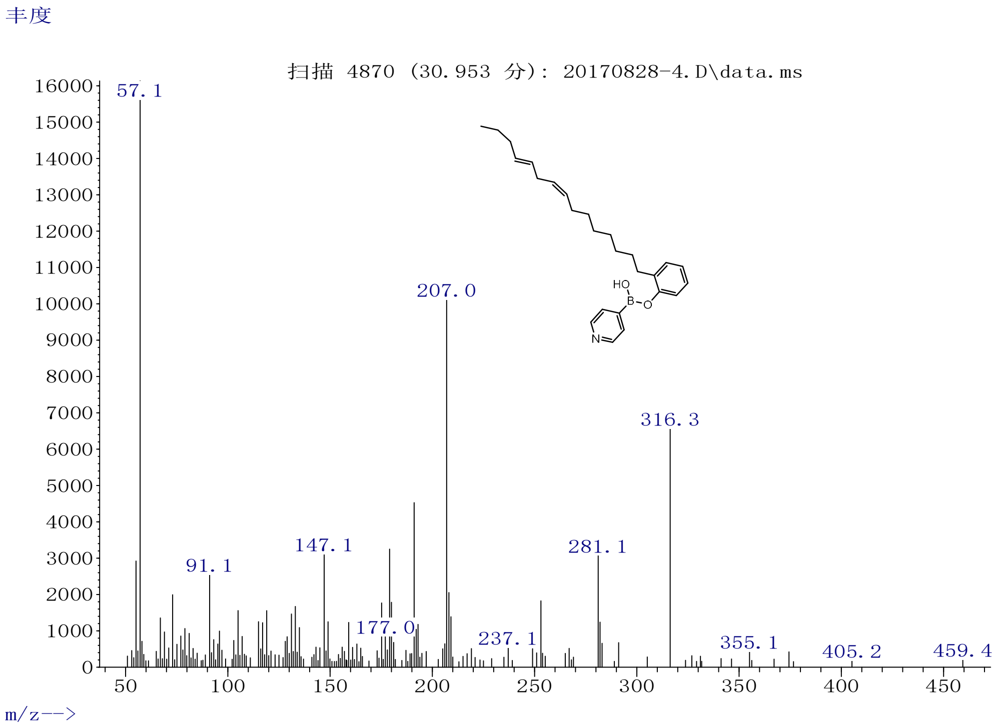

MS Compound 16 S5-3

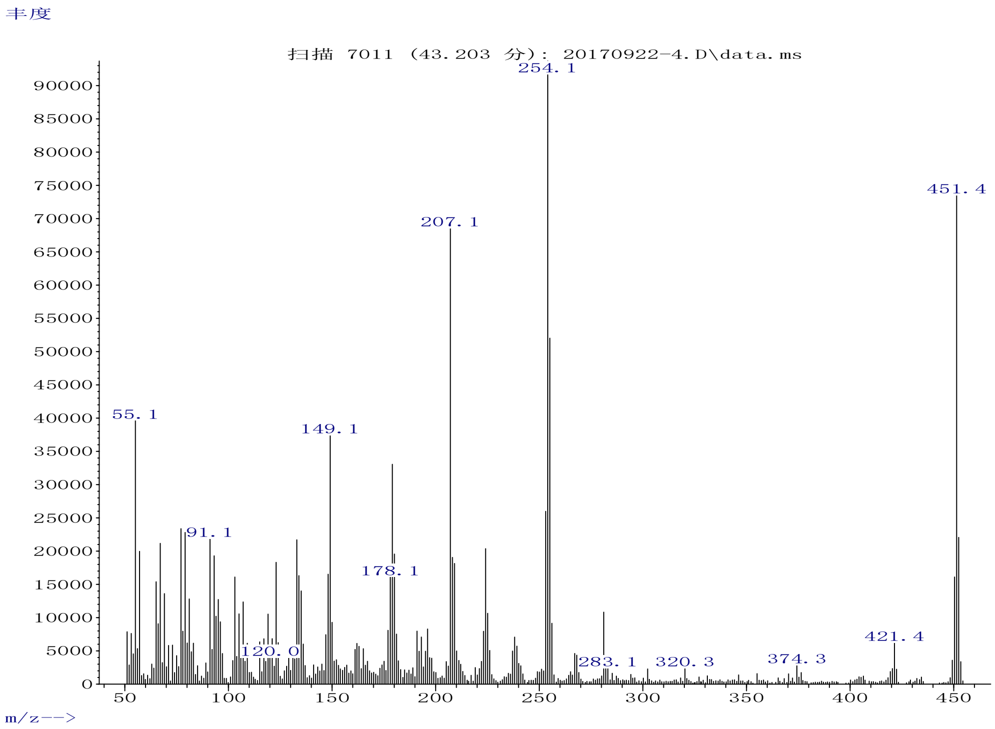

NMR 1H/13C Compound 1 T5-3

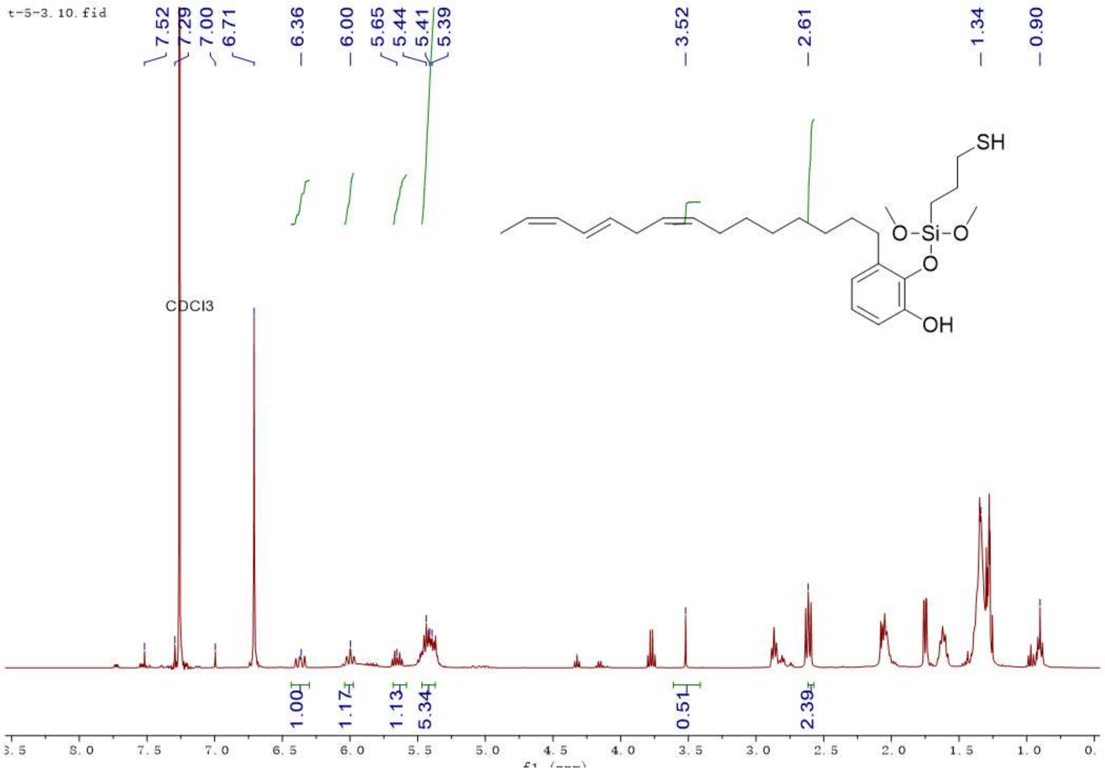

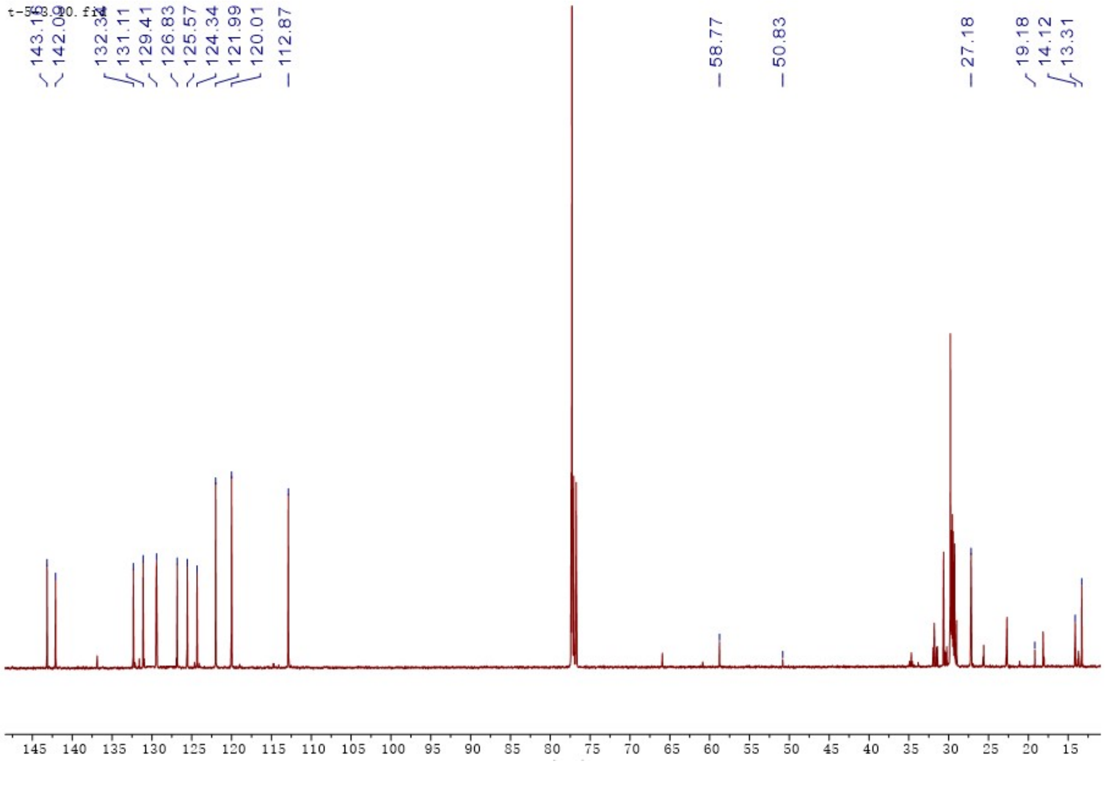

NMR 1H/13C Compound 2 T5-5

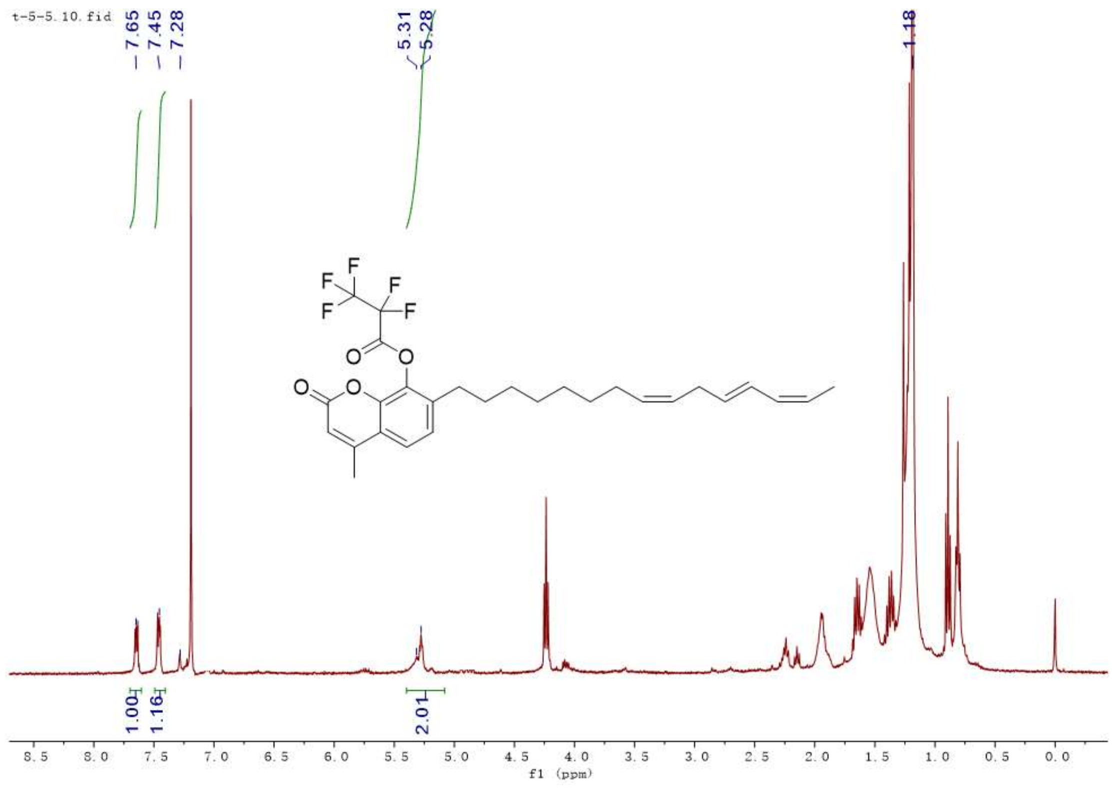

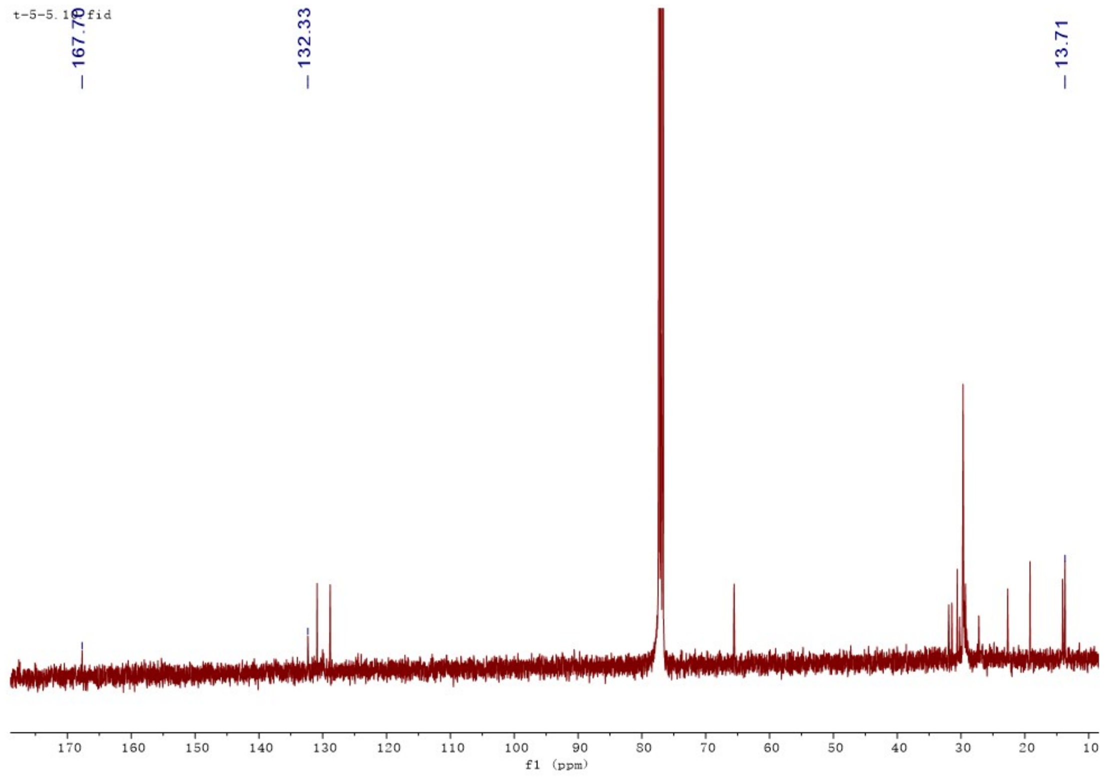

NMR 1H/13C Compound 3 F2-1

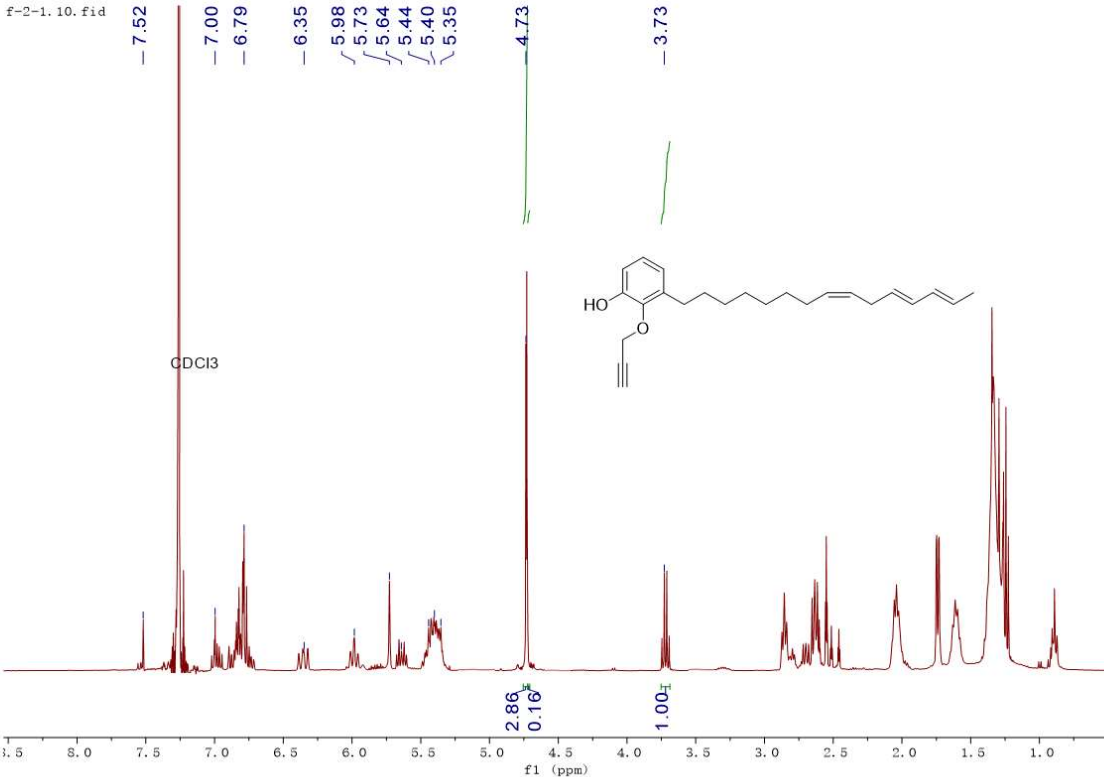

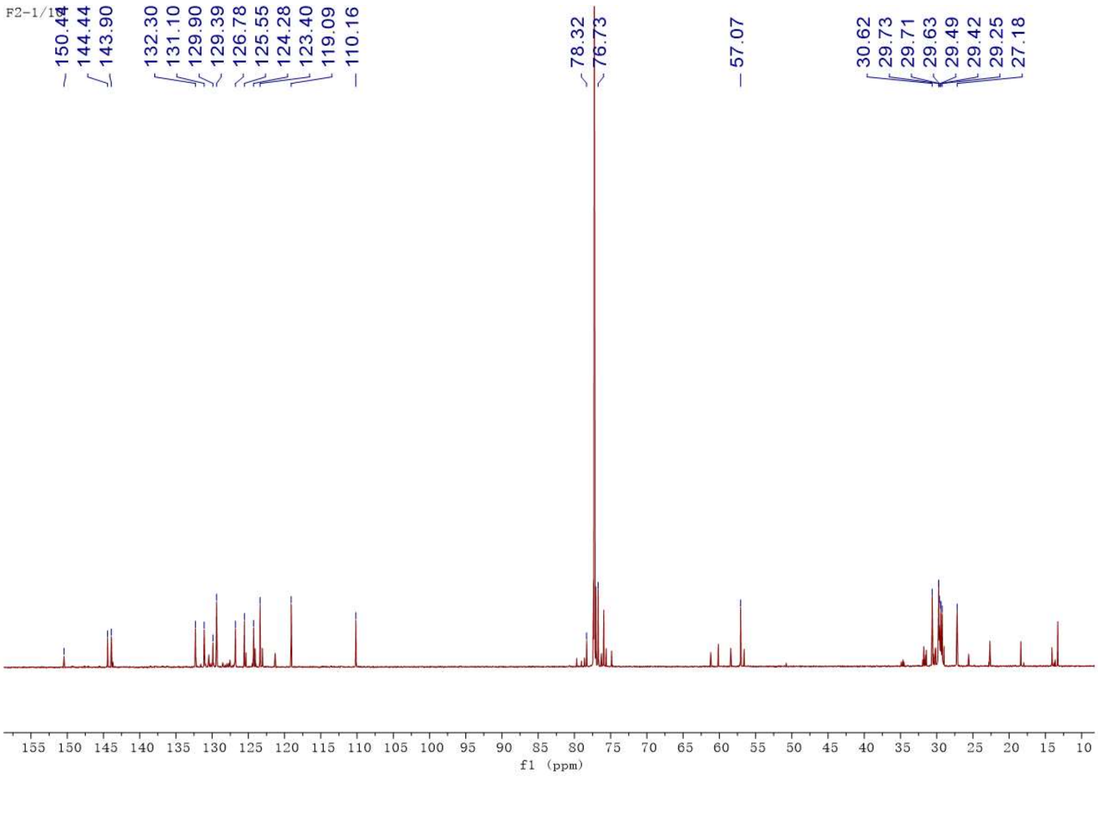

NMR 1H/13C Compound 4 X3-6

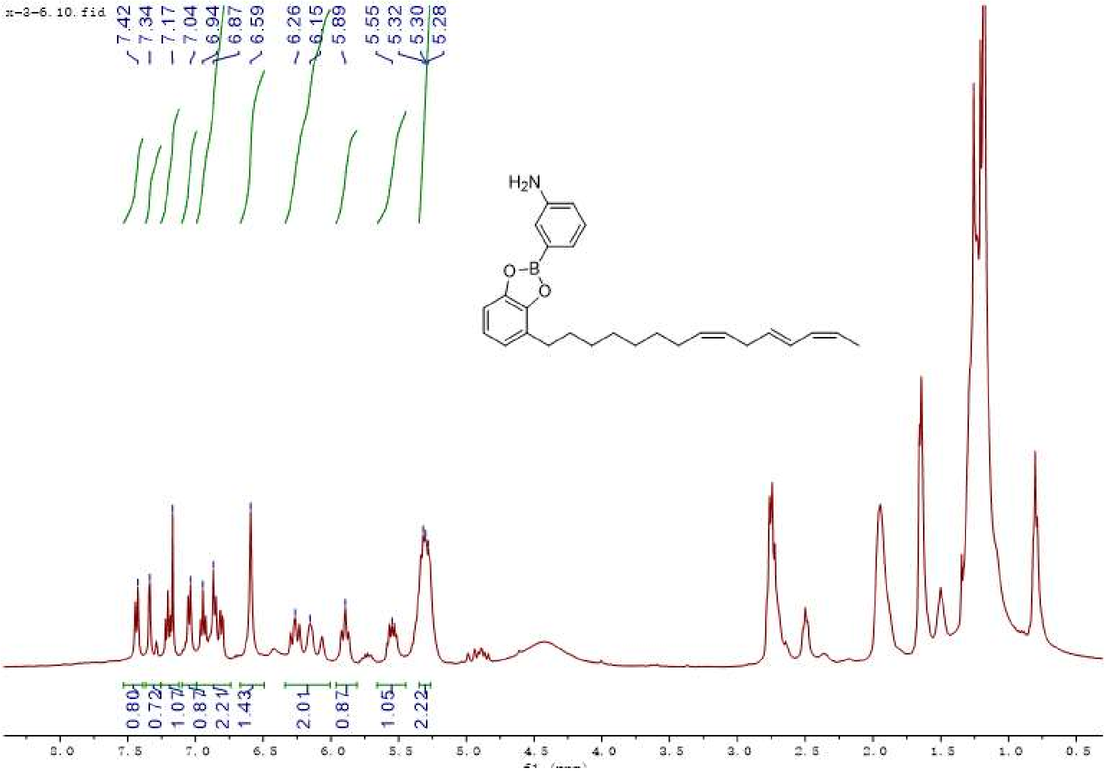

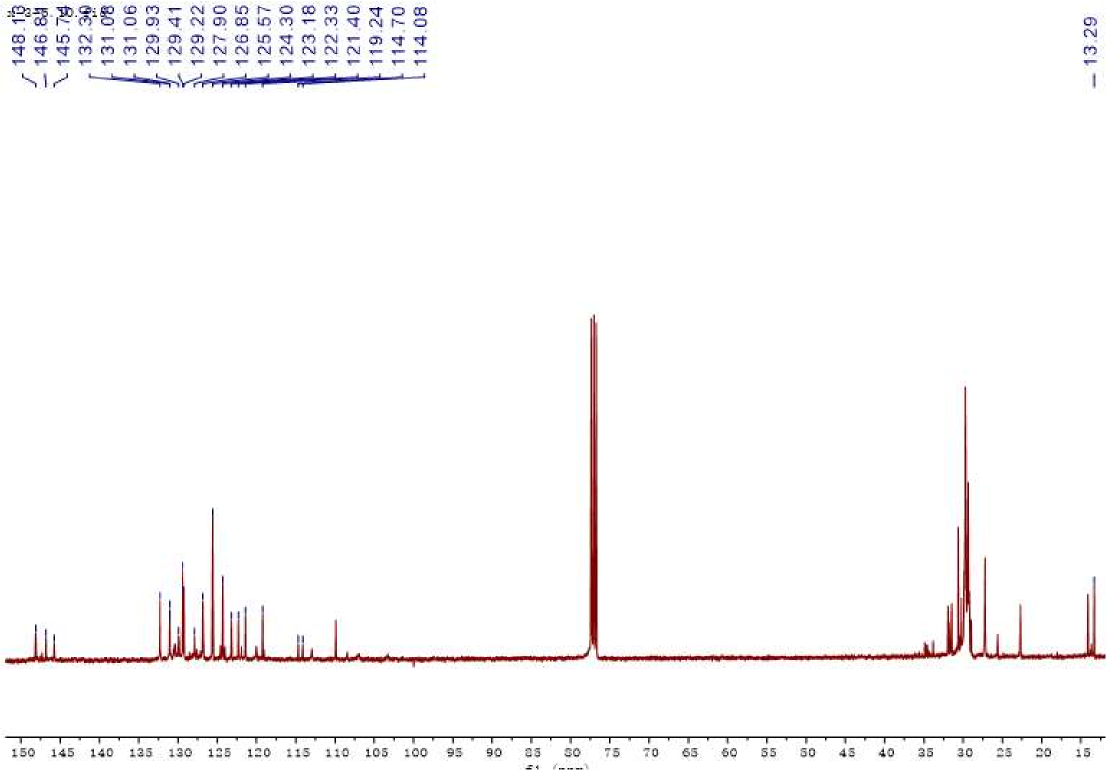

NMR 1H/13C Compound 5 X3-3

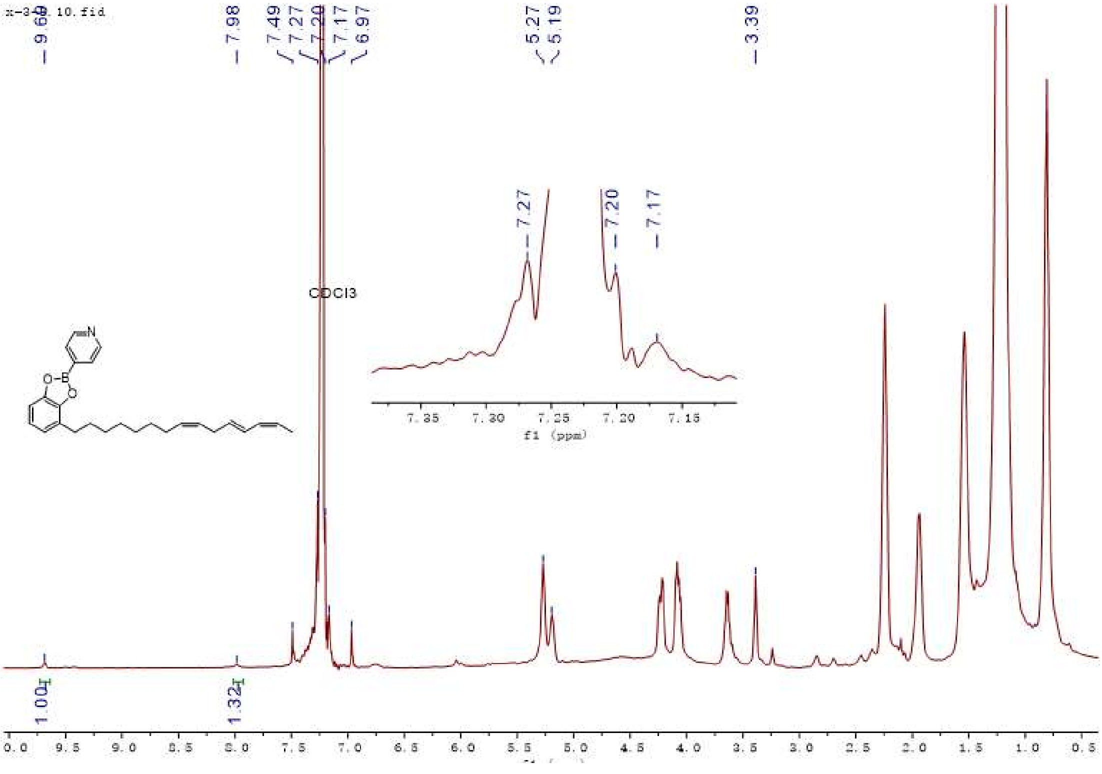

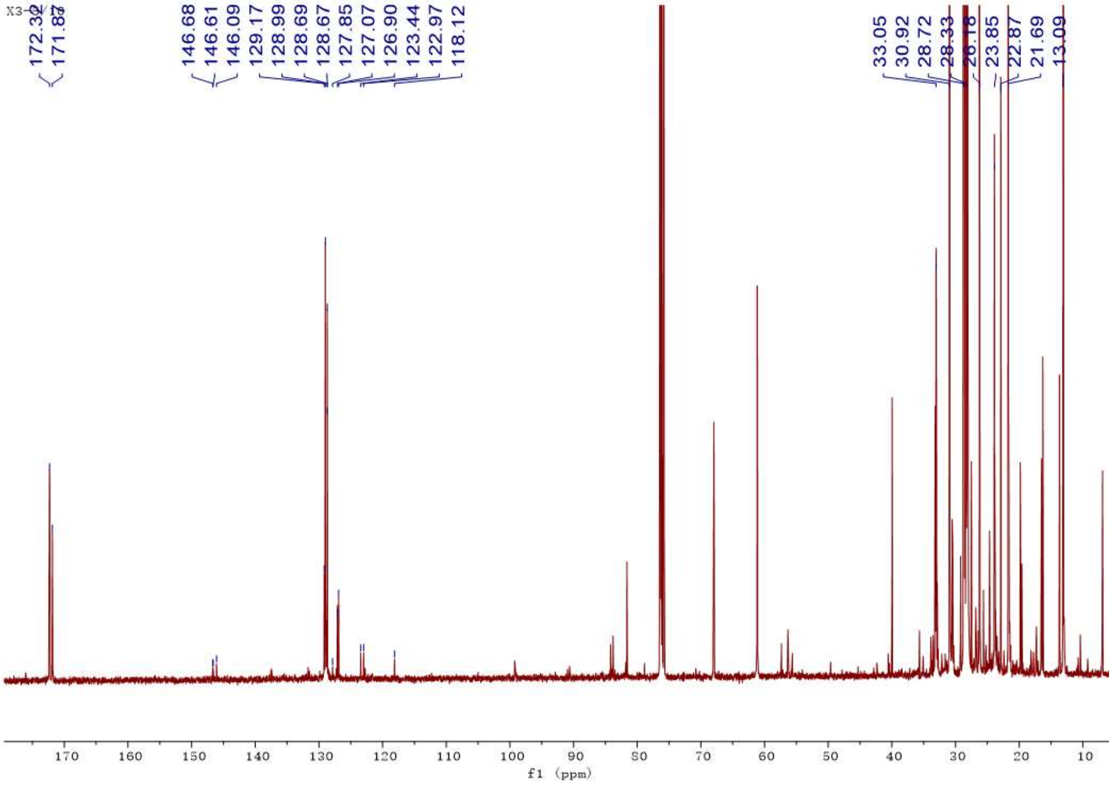

NMR 1H/13C Compound 6 X3-5

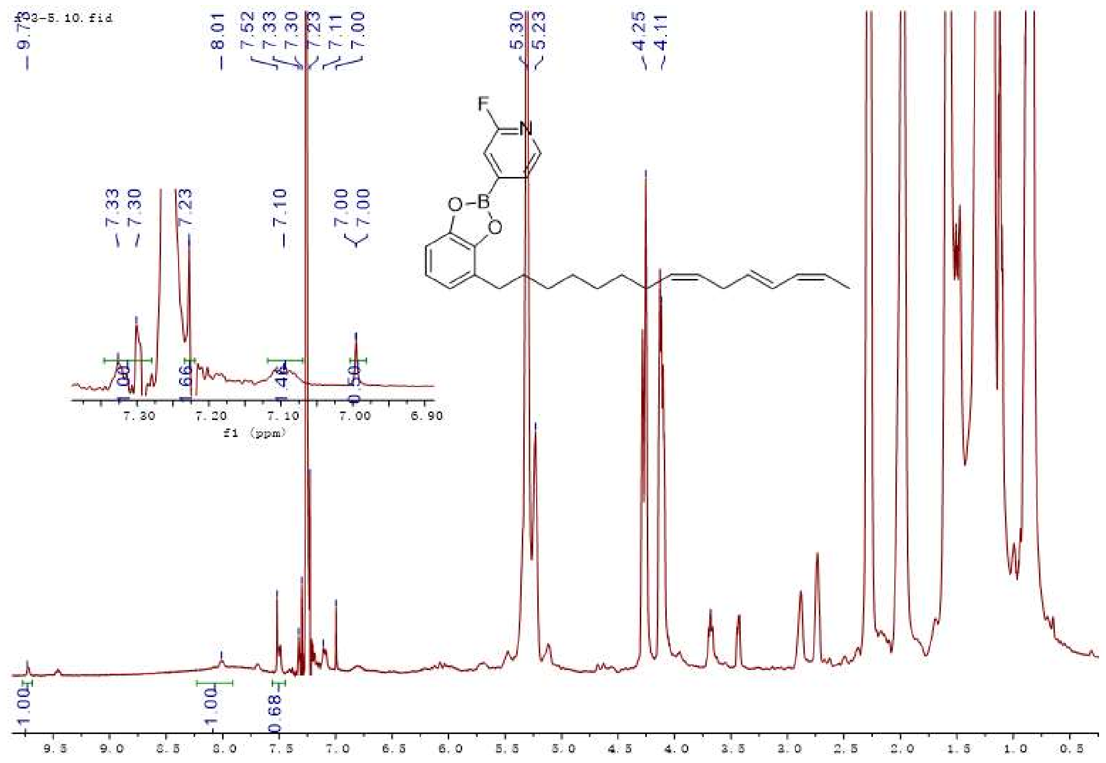

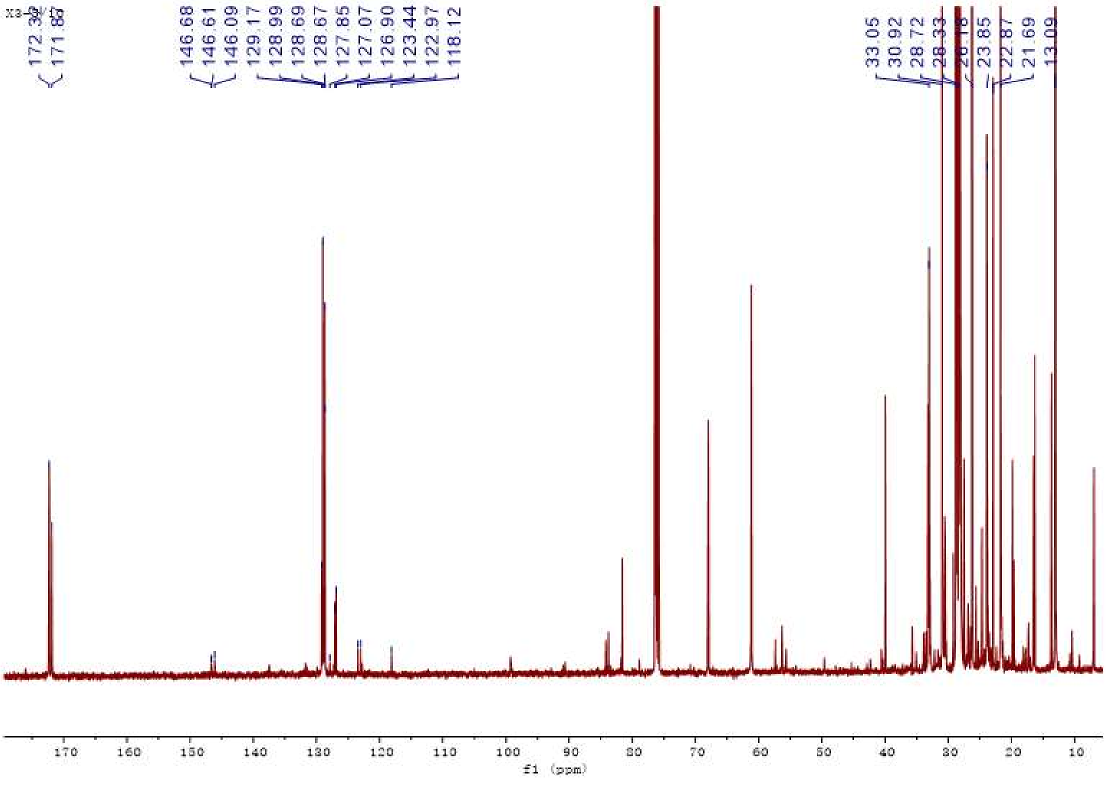

NMR 1H Compound 7 N5-1

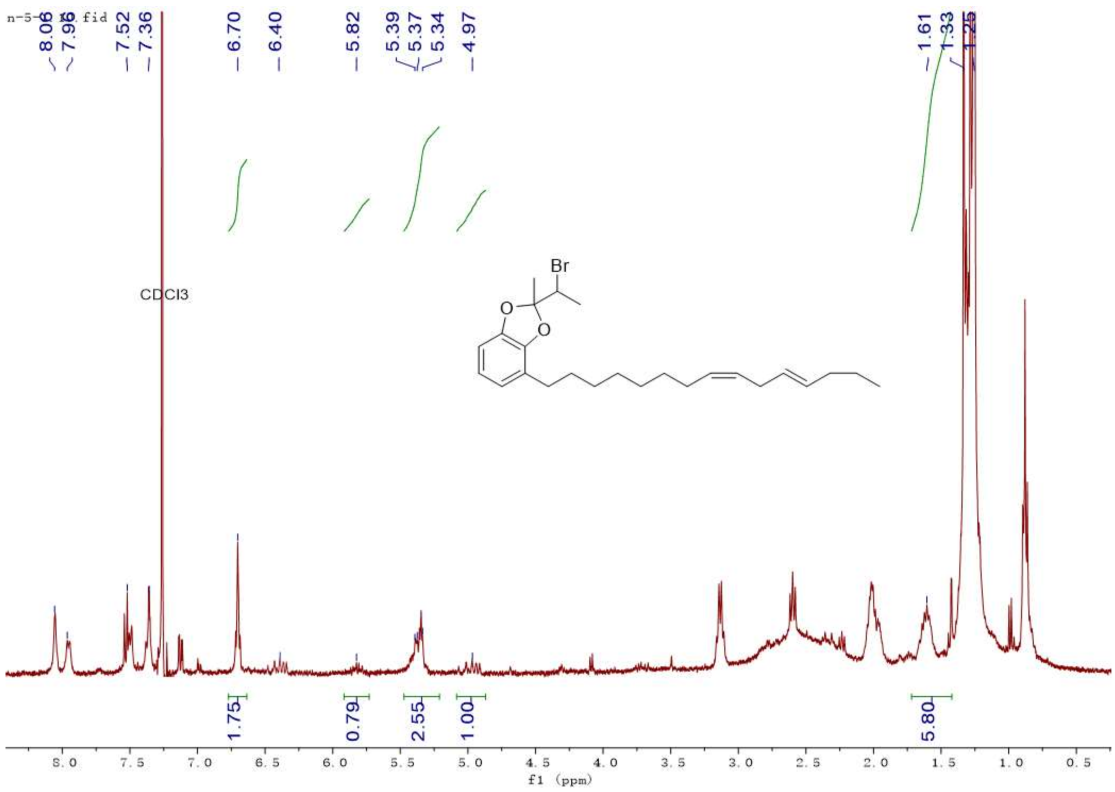

NMR 1H/13C Compound 8 L5-1

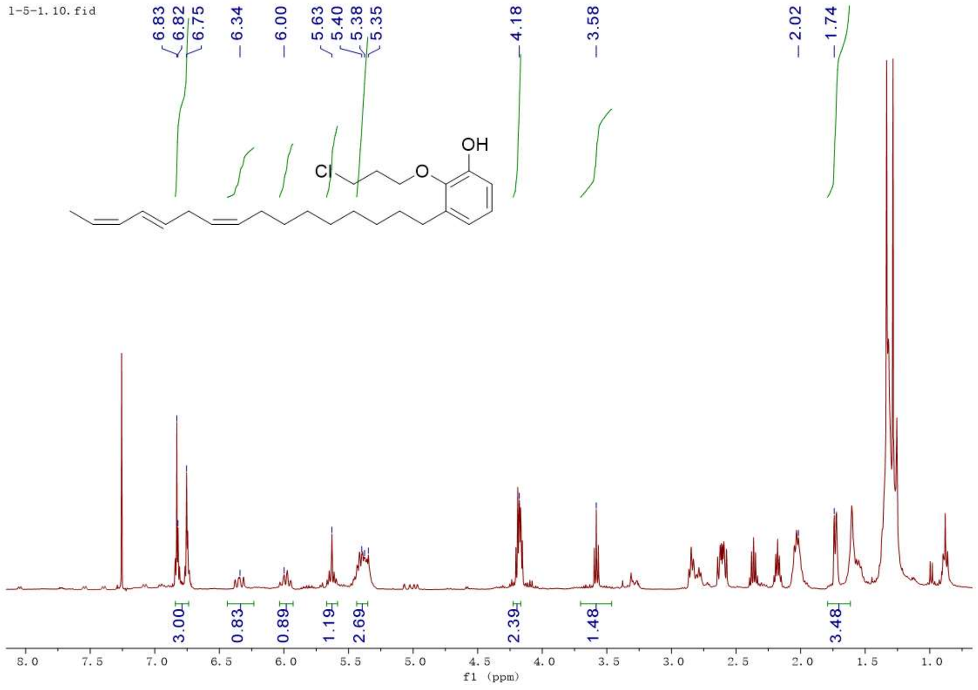

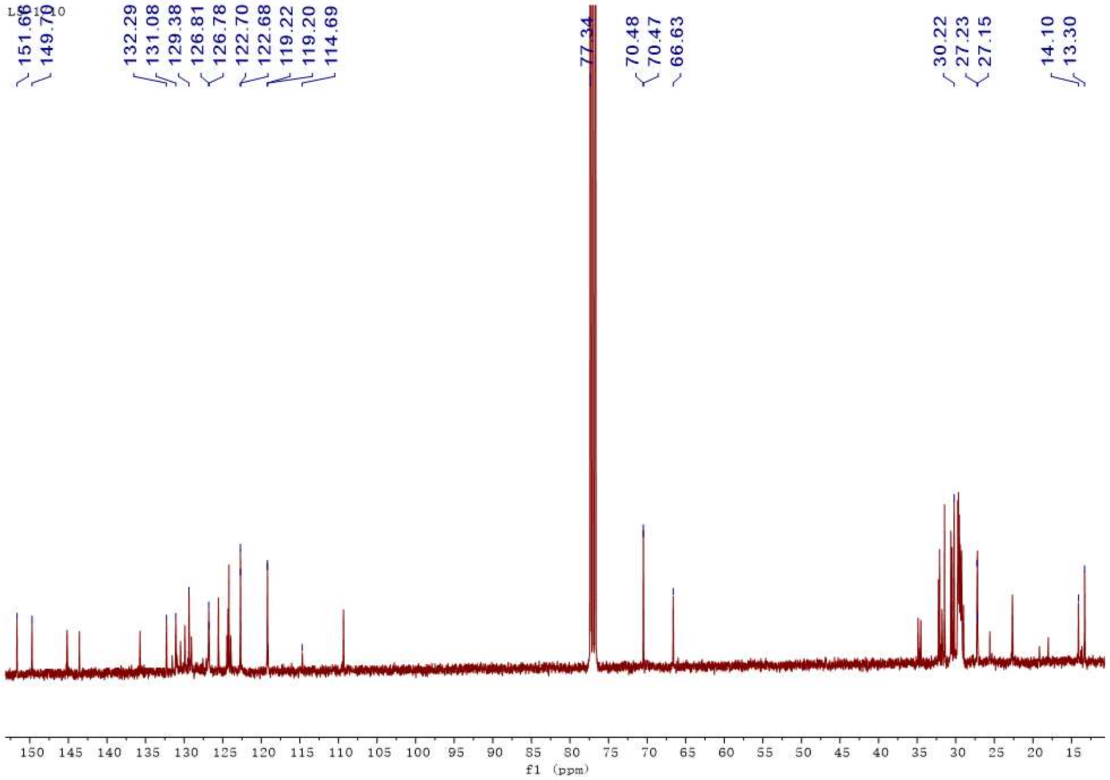

NMR 1H/13C Compound 9 M5-3

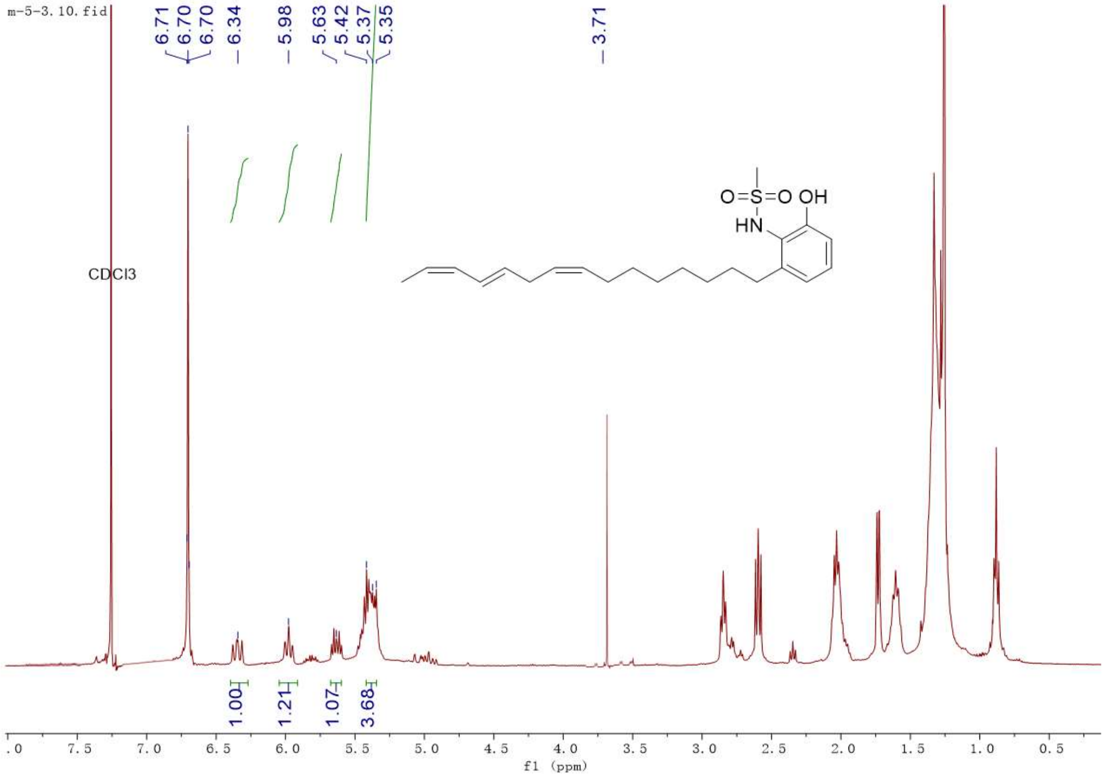

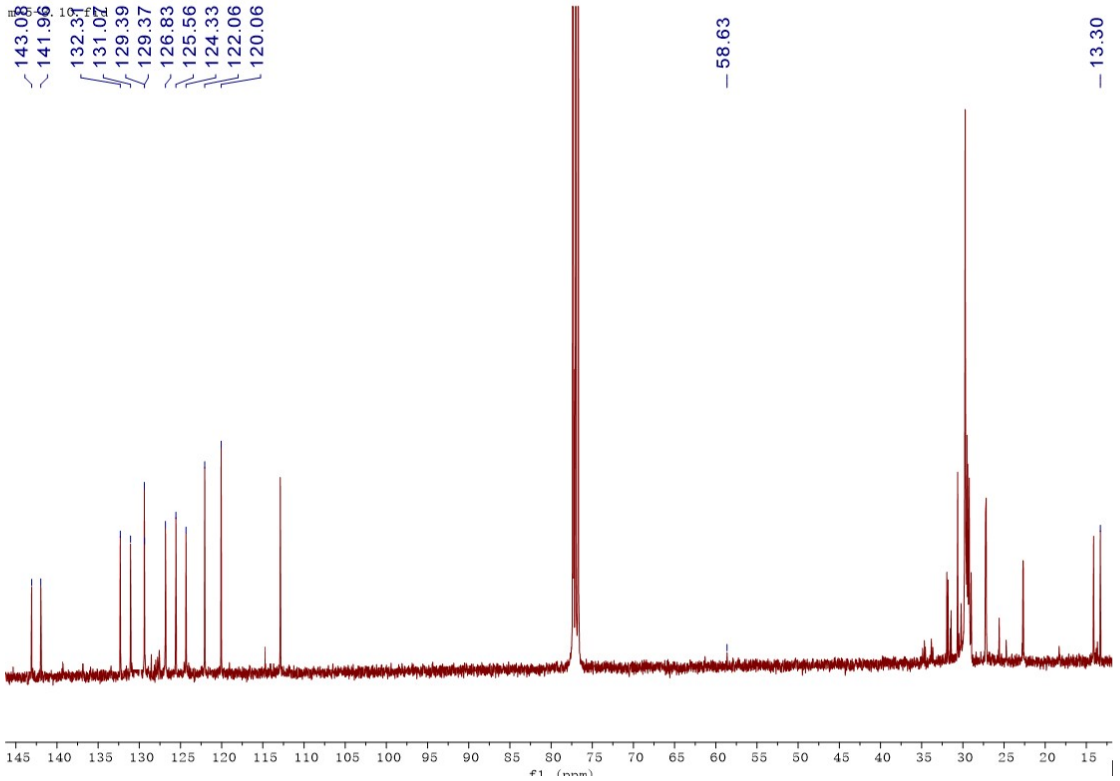

NMR 1H/13C Compound 10 N2-1.(8-hydroxy-4-methyl-7-((8Z,11E,13Z)-pentadeca-8,11,13-trien-1-yl)-2H-chromen-2-one)

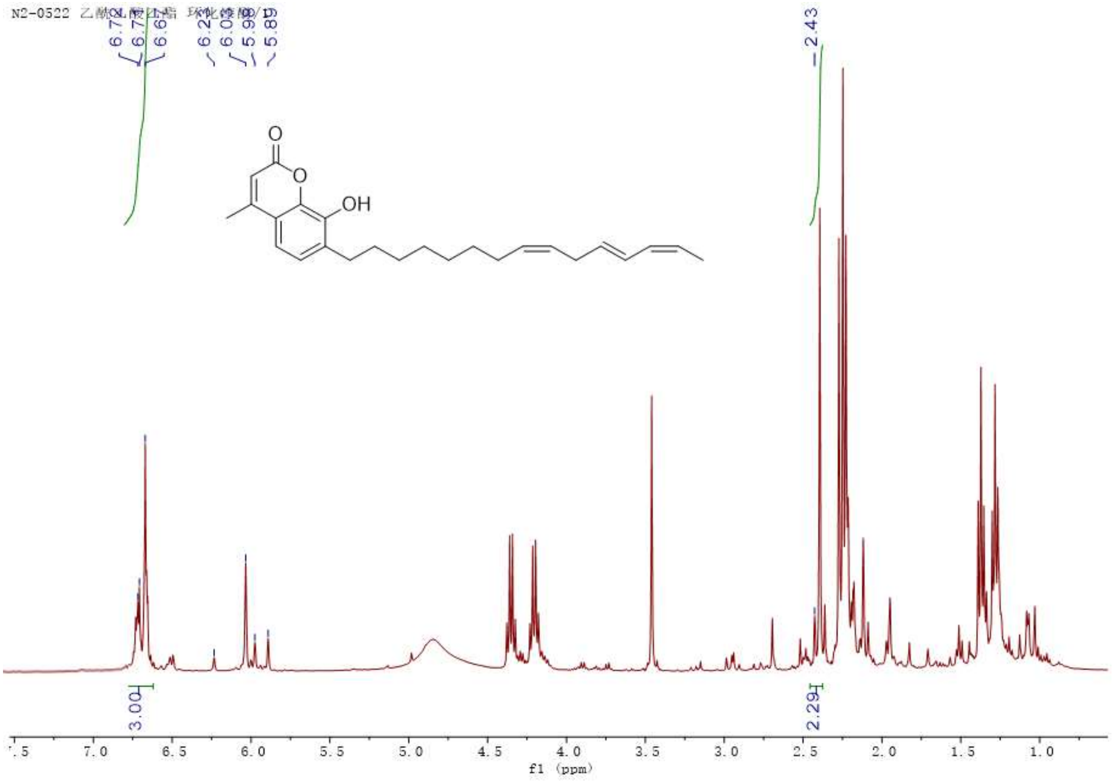

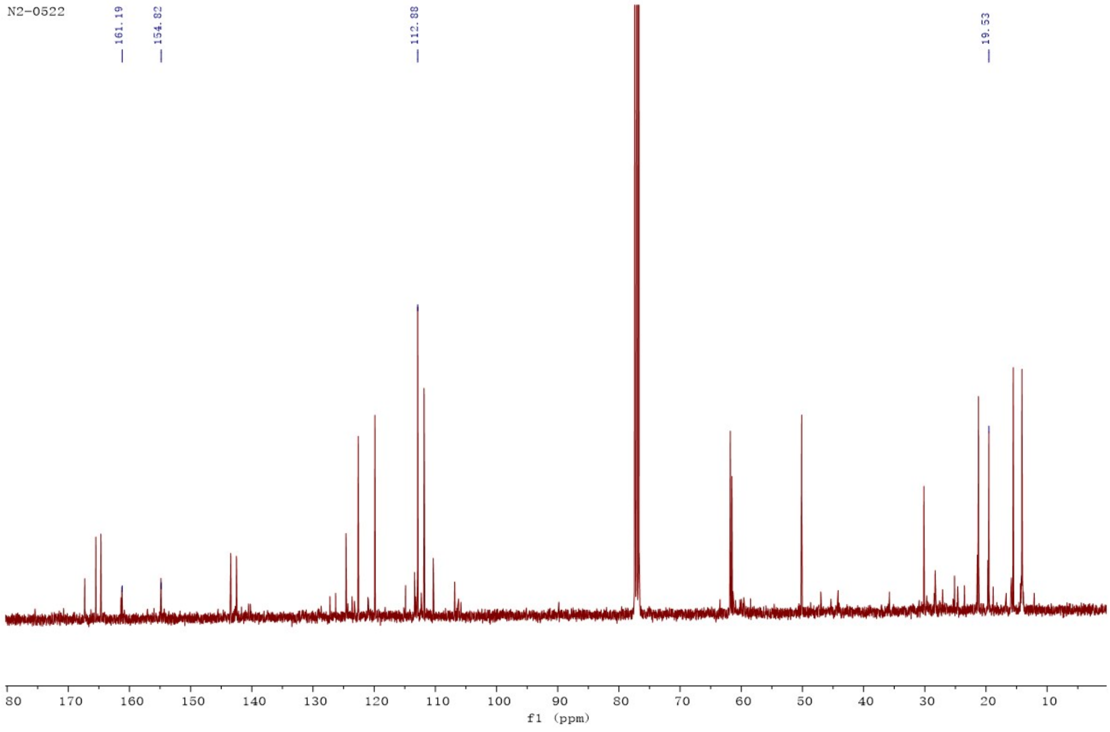

NMR 1H Compound 11 T5-6

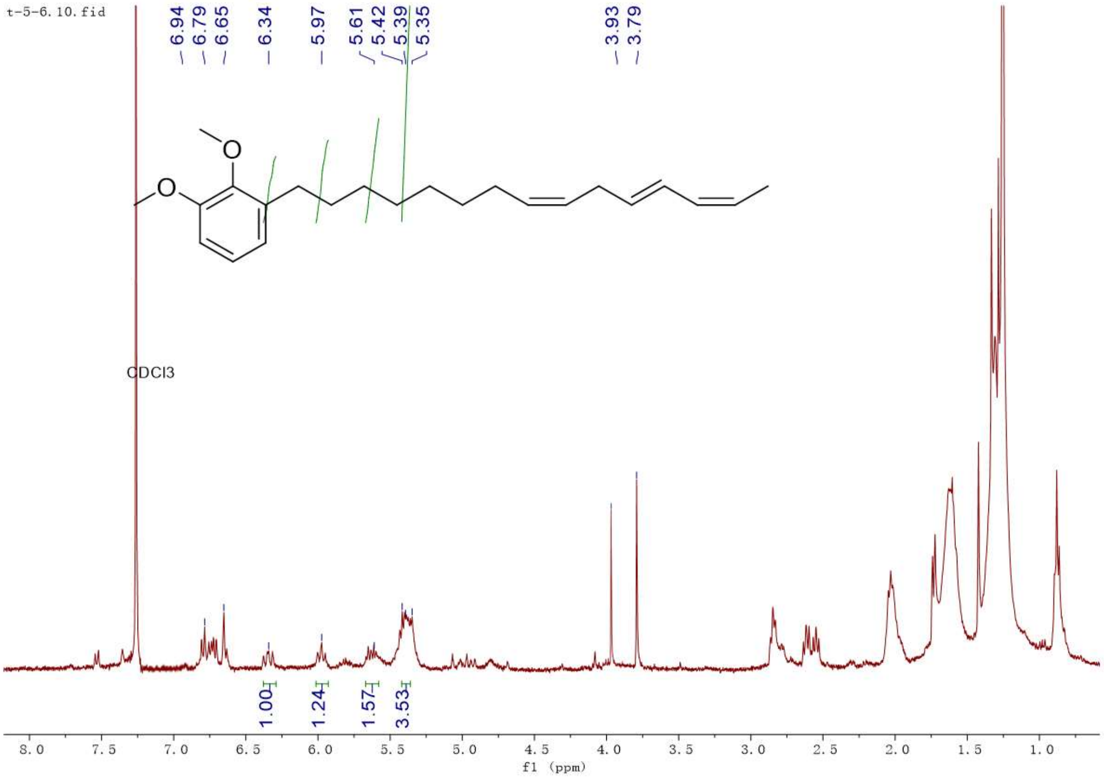

NMR 1H/13C Compound 12 N2-1. (8-hydroxy-4-methyl-7-((8Z,11E,13Z)-pentadeca-8,11,13-trien-1-yl)-2H-chromen-2-one)

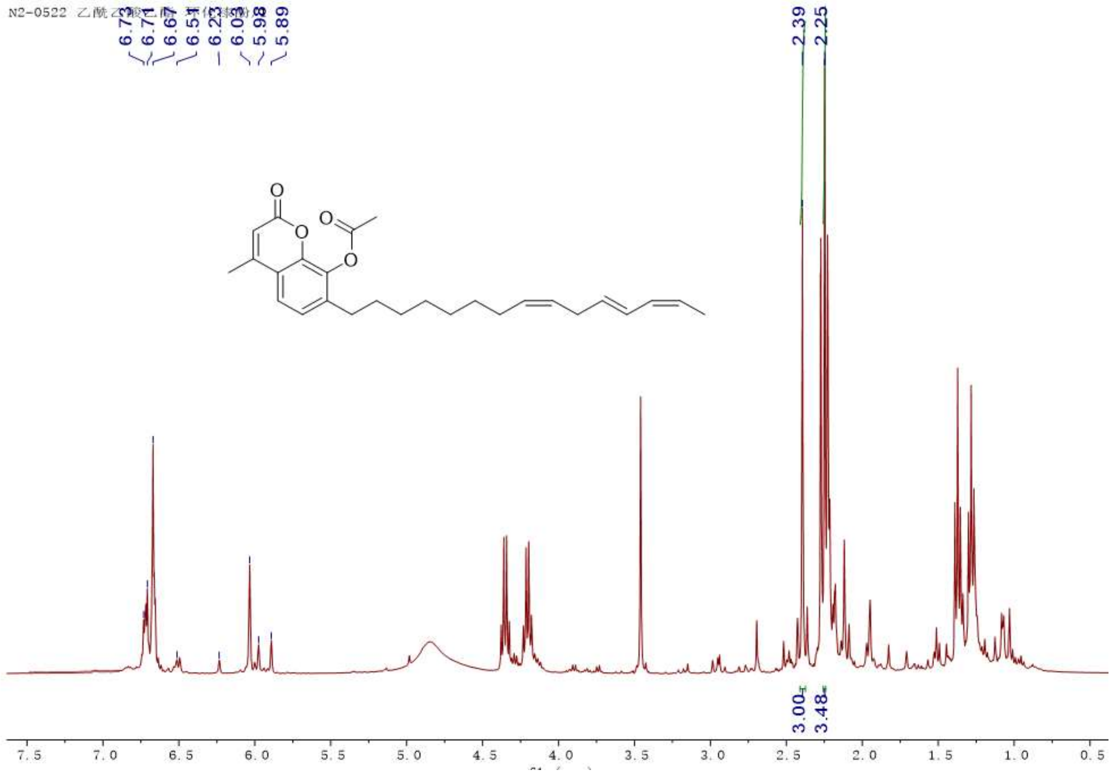

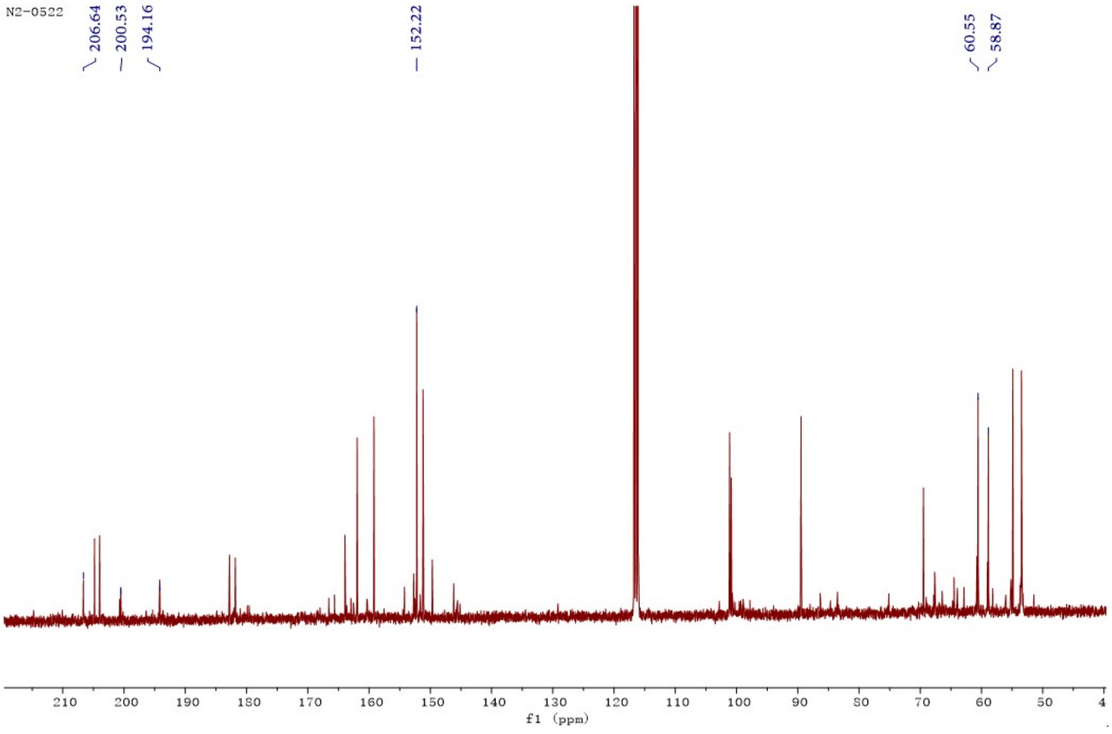

NMR 1H/13C Compound 13 S5-1

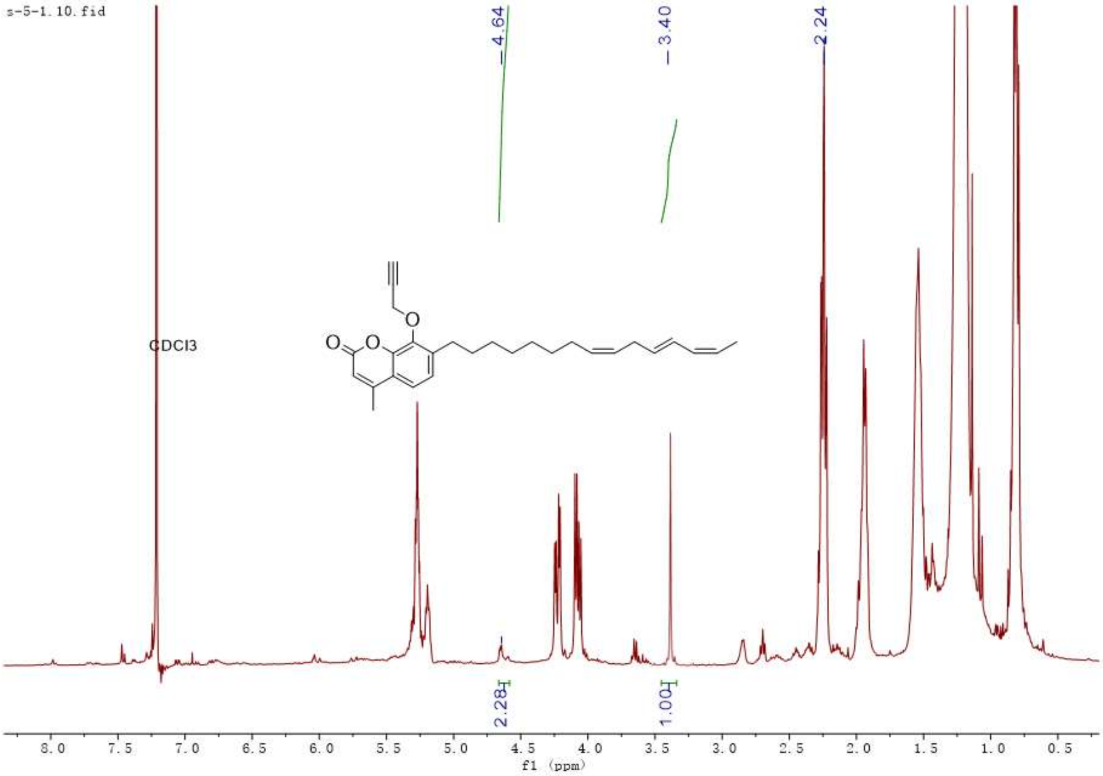

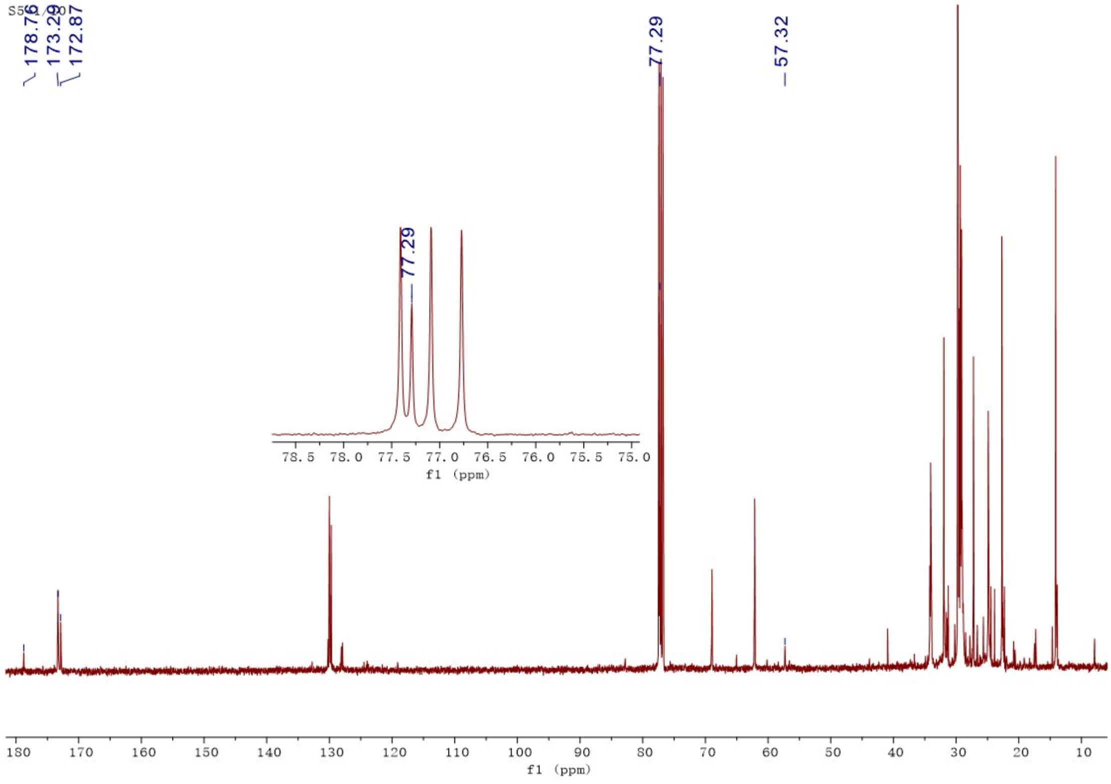

NMR 1H Compound 14 S5-2

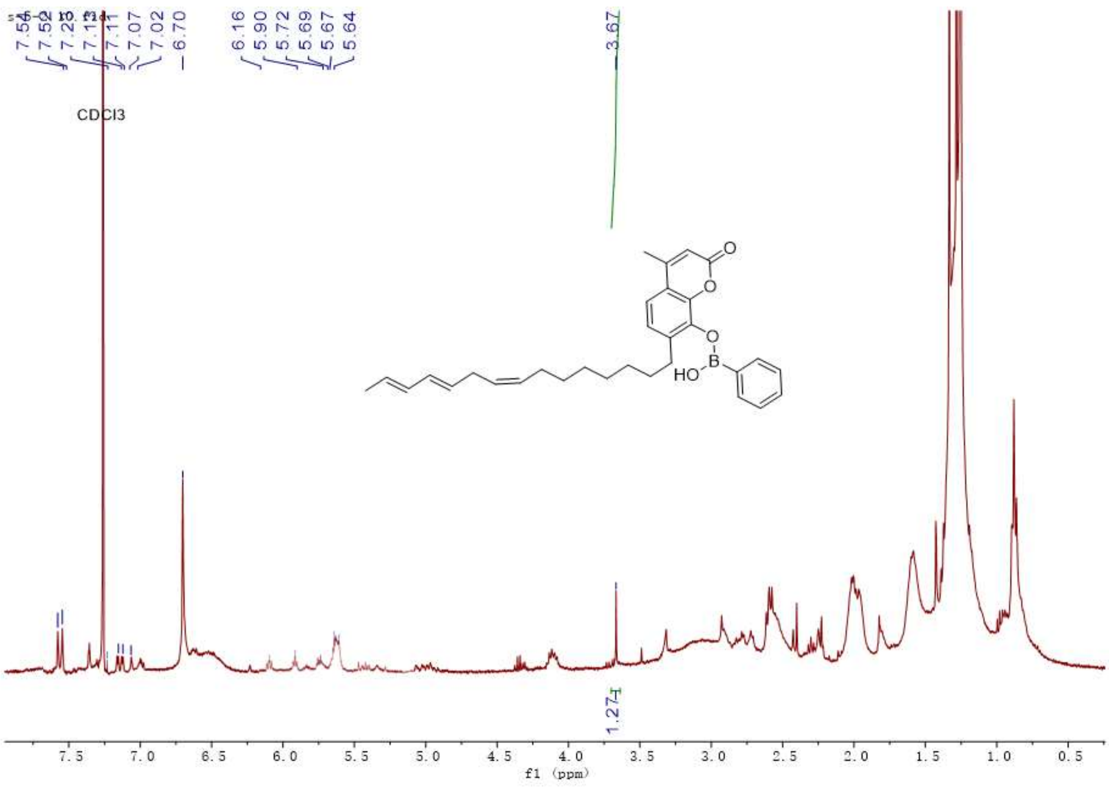

NMR 1H Compound 15 S5-3

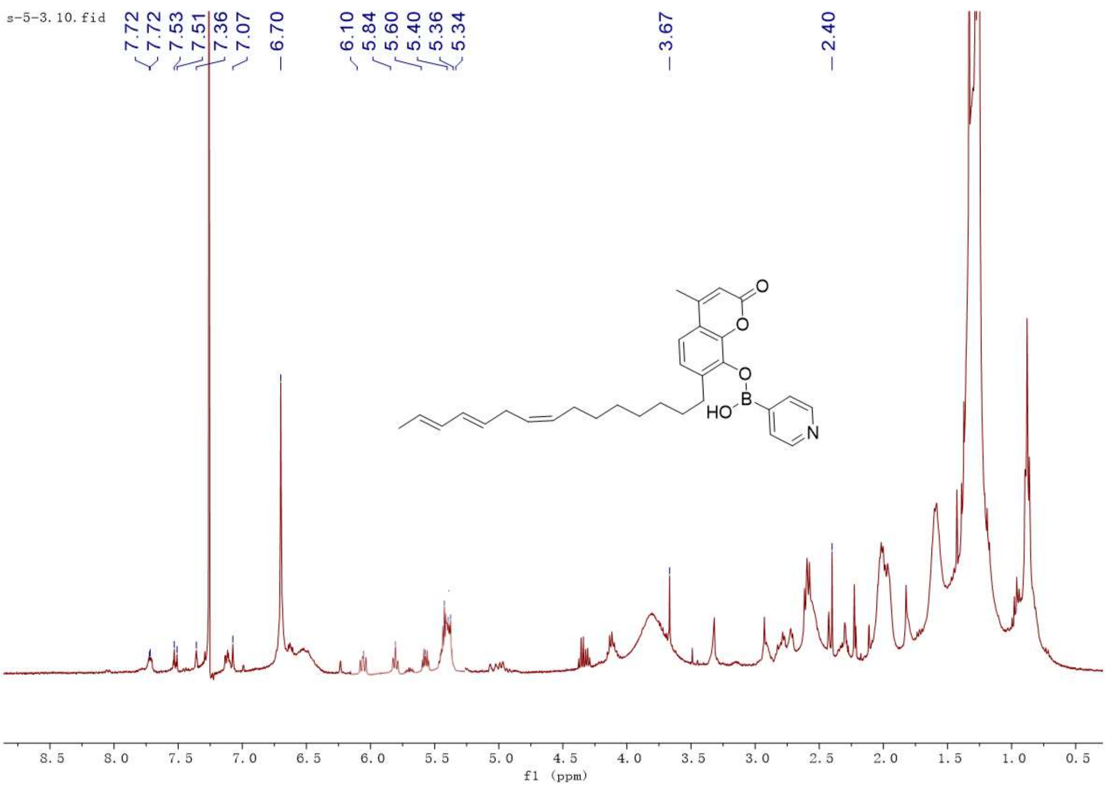

NMR 1H Compound 16 S5-4

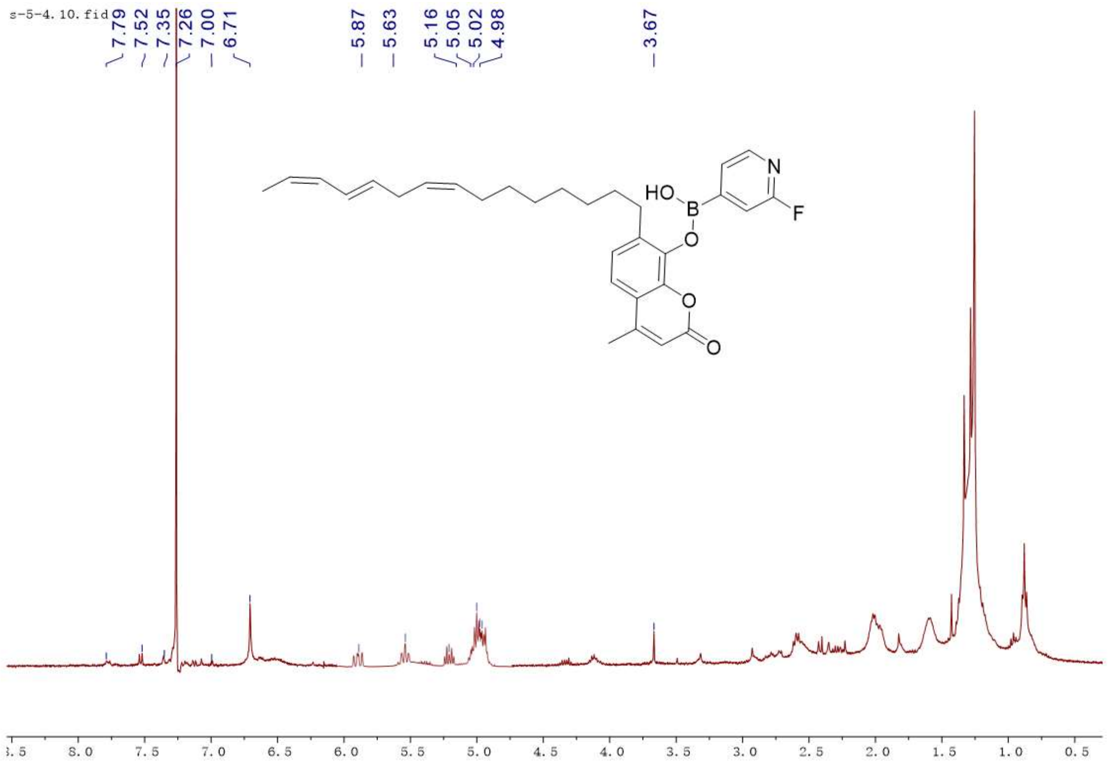

Supplement: Supplementary file 1 [file molecules-23-01074-s001.pdf]
